# The mitochondrial genomes of the endemic and endangered South Philippine Hawk-eagle *Nisaetus pinskeri* and other *Nisaetus* Hawk-eagles (Aves, Accipitriformes)

**DOI:** 10.1371/journal.pone.0360538

**Published:** 2026-10-26

**Authors:** Dhan Mikhail Perdon, John Michael Egana, Francis Tablizo, Renato Jacinto Mantaring, Kris Punayan, Shiela Mae Araiza, Jo-Hannah Llames, Ma. Celeste Abad, Jayson Ibañez, Cynthia P. Saloma

**Affiliations:** 1 National Institute of Molecular Biology and Biotechnology, University of the Philippines Diliman, Quezon City, Philippines; 2 Philippine Genome Center, University of the Philippines Diliman, Quezon City, Philippines; 3 Philippine Eagle Foundation, Philippine Eagle Center, Davao City, Philippines; Instituto Leonidas e Maria Deane / Fundacao Oswaldo Cruz, BRAZIL

## Abstract

Southeast Asia is home to 22 endemic raptors, and almost all are classified as “Endangered” or higher by the International Union for Conservation of Nature and Natural Resources (IUCN). The limited range and fragmented distribution of some Southeast Asian raptors from the genus *Nisaetus* hinder the characterization of these endangered birds. This study presents the mitochondrial sequences of the Pinsker’s Hawk-eagle (*Nisaetus pinskeri*) and Changeable Hawk-eagle (*Nisaetus cirrhatus*) and conducts a comparative mitogenome analysis across Accipitriformes. The partial mitogenome lengths of *N. pinskeri* and *N. cirrhatus* are 18,654 bp and 17,874 bp, with a GC content of 46.4% and 47.4%, respectively. An extra cytosine at position 174 of the *nad3* gene was present in both mitogenomes, which was not previously observed in other *Nisaetus* species. Phylogenetic analysis recapitulated the topology of the order Accipitriformes. It showed that *N. pinskeri* and *N. cirrhatus* are closely related to each other and sister to the rest of the *Nisaetus* representatives. Moreover, the trees based on concatenated mitochondrial protein-coding genes were closer to the latest reported phylogenies than single-gene trees using conventional mitochondrial markers. The selection pressure analysis showed that all protein-coding genes experienced purifying selection, with the highest in *atp8* and the lowest in *cox1*. As the first report of the near-complete mitogenomes of these raptors, this study expands the available genomic resources for these understudied raptors and provides a foundation for future population genetic research.

## Introduction

Southeast Asia is home to 22 endemic raptors, with the Philippines having four endemic raptors. The Pinsker’s hawk-eagle or South Philippine hawk-eagle (*Nisaetus pinskeri*) is an endangered raptor endemic to the southern islands of the Philippine archipelago. It is the second most threatened raptor in the Philippines, next to the Philippine Eagle (*Pithecophaga jefferyi*). It lives in primary lowland forests and is threatened by habitat loss and other human activities [1]. Today, there are an estimated 600–800 mature adults with a decreasing population trend, resulting in an “Endangered” classification from the International Union for Conservation of Nature (IUCN) Red List of Threatened Species [1]. On the other hand, the Changeable hawk-eagle (*Nisaetus cirrhatus*) has an extensive range stretching from the Indian subcontinent to the Southeast Asian islands with several subspecies. It is currently classified as Least Concern, as this hawk-eagle remains resilient in the face of deforestation and habitat destruction, partly due to its adaptability to many habitat types [2,3].

Hawk-eagles of the genus *Nisaetus* are part of the family Accipitridae, one of the largest groups of non-passerine birds, containing 256 species in 75 genera. Together with its sister genus *Spizaetus*, *Nisaetus* hawk-eagles have a prominent head crest as their defining feature [4]. These genera were separated when a mitochondrial study by Haring et al. [4], using partial cytochrome b (*cytb*) and control region sequences, showed that *Spizaetus* birds in Asia are genetically distinct from *tho*se in the Americas due to convergent evolution, leading to the reclassification of Spizaetus into the new genus *Nisaetus*. Likewise, *N. pinskeri* was formerly considered a subspecies of the Philippine hawk-eagle (*N. philippensis*) but was elevated to species status following a mitochondrial study by Gamauf et al., also using *cytb* and control region sequences, that demonstrated clear genetic differentiation, likely resulting from an early Pleistocene geographic isolation between the Greater Luzon and Greater Mindanao faunal regions [5,6].

Mitochondrial markers have long been used in elucidating avian phylogeny and monitoring population genetics; however, previous large-scale molecular studies have only relied on a few markers [7]. Complete mitogenomes contain more variable sites and thus should have higher phylogenetic resolution than single mitochondrial genes [8–10]. When compared to nuclear markers, whole mitogenomes retain phylogenetic information more consistently because of their physical and evolutionary characteristics [11]. Nevertheless, the maternal inheritance and lack of recombination of mtDNA mean that mitogenomes represent a single evolutionary history, which may not always reflect species relationships [12].

The mitochondrial genomes of birds are usually 16–20 kb in length and contain 13 protein-coding genes, 22 transfer RNAs, 2 ribosomal RNAs, and a control region. A duplicated putative control region between *trnE(ttc)* and *trnF(gaa)* is present in raptors of Accipitriformes [13]. The mitogenome has a highly conserved gene content but evolves about 10 times faster than nuclear genomes, albeit mostly point mutations [14].

The assembly of mitogenomes also provides a reference for genetic diversity assessment, which is an important prerequisite for most conservation strategies. For instance, in the case of the Great Hornbill (*Buceros bicornis*) in Thailand, Jansamut et al. proposed an active genetic management framework incorporating the mitogenome haplotype diversity of threatened populations to choose individuals for reintroduction programs [15]. Additionally, since mitochondria play a crucial role in energy metabolism, analyzing the selection pressure on mitochondrial protein-coding genes can provide insights into how organisms adapt to their environment. In the case of the Peninsular Hill Trout (*Lepidopygopsis typus*), a relaxation of selection was observed, which is a concerning finding with potential implications for the species’ long-term viability [16].

In this study, we assembled the mitochondrial genomes of *Nisaetus pinskeri* and *Nisaetus cirrhatus* and conducted comparative mitogenome analysis with other published data across multiple taxonomic levels. Specifically, we examined nucleotide composition and codon usage, constructed maximum-likelihood phylogenetic trees, and assessed nucleotide diversity and selection pressure among protein-coding genes. The resulting mitogenomes expand the genetic resources available for these species and provide a basis for future studies of their evolutionary history and population genetics.

## Methodology

### Sample collection and DNA extraction

Blood samples of *Nisaetus pinskeri* were collected from captive birds at the Philippine Eagle Center in Malagos, Davao City. The Philippine Eagle Foundation has a 10-year Memorandum of Agreement (MOA) with the Philippine Department of Environment and Natural Resources (DENR), which provides the legal and ethical basis to undertake annual physical and medical screening of raptors as well as undertake genetic studies, species rehabilitation, captive-breeding, and species reintroduction (MOA No. SENR067470). The blood was drawn from the brachial vein on the wing and stored in K2 EDTA tubes to prevent clotting. The samples were transported on ice and stored at 4°C before DNA extraction. DNA extraction was performed using the DNeasy Blood and Tissue Kit (Qiagen) following the manufacturer’s protocol. The sample’s DNA integrity, purity, and concentration were checked using agarose gel electrophoresis, NanoDrop Eight Spectrophotometer (ThermoFisher Scientific), and Qubit 2.0 Fluorometer (Life Technologies), and subsequently stored at −20°C. No animals were sacrificed for this study, and no anesthesia or analgesia was required, as venipuncture is a minimally invasive procedure routinely performed during annual veterinary health checks. To minimize stress and discomfort, birds were handled by trained veterinarians and animal keepers following standard welfare protocols, handling time was kept as short as possible, and birds were promptly returned to their enclosures after sampling.

### Library preparation and Sequencing

Whole genome sequencing was performed using the DNA Nanoball Sequencing technique. Approximately 100ng of genomic DNA was used for library preparation using the MGIEasy FS DNA Library Prep Kit (MGI). The genomic DNA was diluted, randomly fragmented, and tagged with adapters. Following library preparation, the final concentration was checked using the Qubit dsDNA BR assay (Life Technologies), and the average library size was determined using the Tapestation 2000 (Agilent Technologies). The library was denatured, circularized, and digested using the MGIEasy Circularization Module (MGI). The final library of single-stranded circular DNA was diluted and transformed into a DNA Nanoball (DNB). The DNB was sequenced on the DNBSEQ-G400 platform, generating 150 bp paired-end reads.

### Mitogenome assembly and annotation

Sequencing reads of *N. pinskeri* were demultiplexed and trimmed of their adapters from sequencing. Publicly available sequencing data of *Nisaetus floris* (SRR19580041) and *N. cirrhatus* (SRR17839678) were downloaded from NCBI GenBank. The sequencing reads were assessed for their quality using Fastp [17], and bases with quality scores less than Q30 were removed. The mitochondrial genomes were assembled using NOVOPlasty [18] with their respective cytochrome oxidase subunit I (*cox1*) genes (MT158255.1 for *N. cirrhatus* and AY700125.1 for *N. pinskeri*) from NCBI GenBank as seed sequences. The complete mitogenomes of *Nisaetus alboniger* (NC_007599.1) and *Nisaetus nipalensis* (NC_007598.1) were also obtained from NCBI GenBank and reanalyzed.

Mitogenome assembly of *N. pinskeri* with NOVOPlasty resulted in two contigs. The inspection of *N. pinskeri* contigs revealed an overlap with similar repeat regions at the end of contig 1 and the start of contig 2. The two contigs were manually resolved by concatenation while considering all 24 instances of repeat regions in both contigs. On the other hand, assembly of *N. cirrhatus* resulted in one circularized assembly.

Additional assemblies were generated using MitoZ [19], MitoFinder [20], and GetOrganelle [21] (S1 Fig). Mitogenome assemblies generated by MitoZ and MitoGinder failed to recover the gene block consisting of *nad6, trnP,* and *trnE*, located between the control region and pseudo-control region, and encoded on the negative strand. GetOrganelle recovered all expected avian mitochondrial genes; however, only three repeat units were assembled within the duplicated control-region-associated repeat region, compared to 24 repeat units recovered by NOVOPlasty. The repeat motif recovered in *N. pinskeri* was highly similar to those observed in *N. alboniger* and *N. nipalensis* (S2A Fig), which contain 19 and 13 repeat units, respectively. Given the recovery of the complete gene complement and repeat architecture consistent with related *Nisaetus* species, the NOVOPlasty assembly was selected for all downstream analyses.

The mitogenomes were annotated using MITOS2, and the circular map was visualized using CGView in the Proksee web tool [22]. GC content and GC skew were also calculated and visualized in Proksee using a 500-bp sliding window with a step size of one nucleotide. Predicted protein-coding genes and their start and stop codons were verified using the ExPASy-Translate Tool [23] with the vertebrate mitochondrial genetic code, while predicted transfer RNAs (tRNAs) were verified using tRNAscan-SE v2.0 [24].

To evaluate sequencing coverage across the mitogenomes, short reads were mapped back to their respective assembled reference sequences using Minimap2 [25] with the short-read alignment preset (-ax sr). For *N. nipalensis* and *N. alboniger*, publicly available sequencing runs DRR190839 and SRR21034932, respectively, were used. Alignment files were converted to BAM format, sorted, and indexed using SAMtools vX [26]. Alignment statistics were summarized using the SAMtools flagstat function, while per-base sequencing depth was calculated using SAMtools depth with the -a option to retain positions with zero coverage. Mean and minimum sequencing coverage were calculated from the per-base depth files. Breadth of coverage and regional coverage statistics were additionally assessed using SAMtools coverage. Per-base read depth was visualized using R and ggplot2.

### Comparative mitogenome analyses

Complete mitogenomes of the family Accipitridae were downloaded from the NCBI GenBank database (Table 1) for analysis, along with the newly sequenced mitogenomes of the genus *Nisaetus*. BLAST alignment of the 24 Accipitridae species was performed with *N. pinskeri* as the reference to identify regions of variability in the mitogenome across the family. The sequences were labeled and arranged according to similarity using the BLAST formatter tool in Proksee.

**Table 1:** List of the 25 Accipitriformes and 5 Cathartiformes birds used for comparative analysis and their mitogenome RefSeq accession numbers.

IUCN Category	Subfamily	Species	Length	RefSeq accession
Least Concern	Accipitrinae	*Accipiter gentilis*	18266	NC_011818.1
Least Concern	Accipitrinae	*Accipiter nisus*	18647	NC_025580.1
Least Concern	Accipitrinae	*Accipiter trivirgatus*	18454	NC_045364.1
Least Concern	Accipitrinae	*Accipiter virgatus*	17952	NC_026082.1
Least Concern	Accipitrinae	*Circus melanoleucos*	17749	NC_035801.1
Least Concern	Aegypiinae	*Gyps fulvus*	18094	NC_036050.1
Least Concern	Aquilinae	*Aquila chrysaetos*	17332	NC_024087.1
Least Concern	Aquilinae	*Aquila fasciata*	18513	NC_029188.1
Vulnerable	Aquilinae	*Aquila heliaca*	18067	NC_035806.1
Endangered	Aquilinae	*Aquila nipalensis*	18450	NC_045042.1
Least Concern	Aquilinae	*Nisaetus alboniger*	17977	NC_007599.1
Near Threatened	Aquilinae	*Nisaetus nipalensis*	17667	NC_007598.1
Least Concern	Aquilinae	*Spizaetus tyrannus*	17479	NC_052803.1
Least Concern	Buteoninae	*Butastur indicus*	19059	NC_032362.1
Least Concern	Buteoninae	*Butastur liventer*	19673	NC_032363.1
Least Concern	Buteoninae	*Buteo buteo*	18674	NC_003128.3
Least Concern	Buteoninae	*Buteo hemilasius*	18079	NC_029377.1
Least Concern	Buteoninae	*Buteo lagopus*	18559	NC_029189.1
Least Concern	Buteoninae	*Haliaeetus albicilla*	17719	NC_040858.1
Least Concern	Buteoninae	*Haliastur indus*	19055	NC_066800.1
Least Concern	Buteoninae	*Milvus migrans*	18016	NC_038195.1
Least Concern	Circaetinae	*Spilornis cheela*	18291	NC_015887.1
Least Concern	Circaetinae	*Circaetus pectoralis*	17473	NC_052805.1
Least Concern	Cathartidae	*Cathartes aura*	16779	NC_007628.1
Least Concern	Cathartidae	*Cathartes burrovianus*	19285	NC_063526.1
Least Concern	Cathartidae	*Coragyps atratus*	19329	NC_063525.1
Least Concern	Cathartidae	*Sarcoramphus papa*	19302	NC_063527.1
Vulnerable	Cathartidae	*Vultur gryphus*	16808	NC_058600.1
Least Concern	Pandionidae	*Pandion haliaetus*	17864	NC_008550.1
Endangered	Sagittariidae	*Sagittarius serpentarius*	16773	NC_023788.1

The nucleotide and amino acid composition were calculated in MEGA 11 [20]. Nucleotide bias, or A-T and G-C skew, was calculated as (A-T)/(A + T) and (G-C)/(G + C), respectively, for the mitogenomes of the genus *Nisaetus* [27]. The relative synonymous codon usage (RSCU) was estimated in MEGA 11. The variable sites and nucleotide diversity metrics, Pi and Kimura 2-Parameter, of the 12 protein-coding genes for each taxonomic level were also computed in MEGA 11. The non-synonymous to synonymous substitution rate ratios (Ka/Ks) of each protein-coding gene in the mitogenomes of the family Accipitridae were calculated using the SLAC tool from Datamonkey [28], which requires codon-based multiple sequence alignments and a corresponding phylogenetic tree as input (see Mitogenomic phylogenetic analysis). The graphs were plotted in RStudio.

### Mitogenomic phylogenetic analysis

The complete mitogenomes of the order Accipitriformes were re-annotated using MITOS2 and were verified accordingly. The *nad6, trnP,* and *trnT* genes were excluded from further analysis as they were not consistently recovered across all taxa during re-annotation. The *nad3* gene of some species was predicted to be separate by the annotation software due to a cytosine insertion at position 174 of the gene, and was manually corrected. The predicted protein-coding genes were aligned using the codon-aware alignment program MACSE v2.03 [29], which aligns coding sequences while preserving codon structure and reading frames. The resulting nucleotide alignments were subsequently trimmed using trimAl with the -terminalonly -automated1 settings before maximum likelihood (ML) phylogenetic analysis.

The trimmed, aligned genes were separated and concatenated into three datasets for ML tree construction: (1) 12 protein-coding genes, (2) 2 ribosomal RNAs (*rrnS* and *rrnL*), and (3) all genes combined (12 PCGs and 2 ribosomal RNAs). For the PCG datasets, each gene was partitioned by codon position into first and second codon positions (pos12) and third codon position (pos3) as separate subsets. Ribosomal RNA genes were each treated as a single partition. Model selection was performed independently for each partition using ModelFinder in IQ-TREE2 [30], with the best-fit substitution model chosen under the Bayesian Information Criterion. Partition schemes were further optimized by merging subsets with statistically similar models using the greedy algorithm implemented in ModelFinder. Maximum likelihood trees were inferred under the partitioned models with 1000 ultrafast bootstrap replicates and 1000 SH-aLRT replicates. To assess whether the amino acid-level signal was consistent with the nucleotide-based topology and to evaluate potential substitution saturation at third codon positions, each PCG was also translated using the vertebrate mitochondrial genetic code and analyzed as an amino acid dataset, with model selection restricted to mitochondrial substitution matrices (mtREV, mtZOA, mtMAM, mtART) and general protein models (WAG, LG).

For comparison, ML trees were also constructed for individual genes, including all 12 PCGs and both ribosomal RNA genes. PCG gene trees retained the pos12 and pos3 codon partition scheme, while ribosomal RNA gene trees were run without partitioning. All per-gene analyses used the same model selection procedure. The phylogenetic trees were visualized and annotated in FigTree v1.4.4 [31]. The Turkey vulture (*Cathartes aura*) from the sister order of Cathartiformes was chosen as the outgroup, and the tips were labeled according to order, family, and subfamily. The Generalized Robinson-Foulds distance was computed using the R package TreeDist to quantify the distance between phylogenetic trees derived from multi-locus datasets and those based on single genes [32].

## Results and discussion

### Characterization of *Nisaetus* mitogenomes

Quality control using Fastp yielded a total of 65.09 G raw reads, of which 64.77 G remained after filtering, with a Q30 percentage of 92.96%, providing the basis for subsequent mitochondrial assembly and coverage analyses. The putative mitochondrial genome of *N. pinskeri* is 18,654 bp in length with 13 protein-coding genes (PCGs), 22 transfer RNAs (tRNAs), 2 ribosomal RNAs (2 rRNAs), a control region (CR), and a pseudo control region (*ψ*CR), shown in Fig 1A. Its length is within the range of other raptors (family Accipitriformes) mitogenomes, with the shortest at 17,332 bp and the longest at 19,673 bp (Table 1). However, when compared to the mitogenomes of the two other *Nisaetus* hawk-eagles, *N. nipalensis* (17,656 bp) and *N. alboniger* (17,967 bp), *N. pinskeri* is much longer, which may be due in part to the consideration of all repeat regions [33].

**Fig 1:**
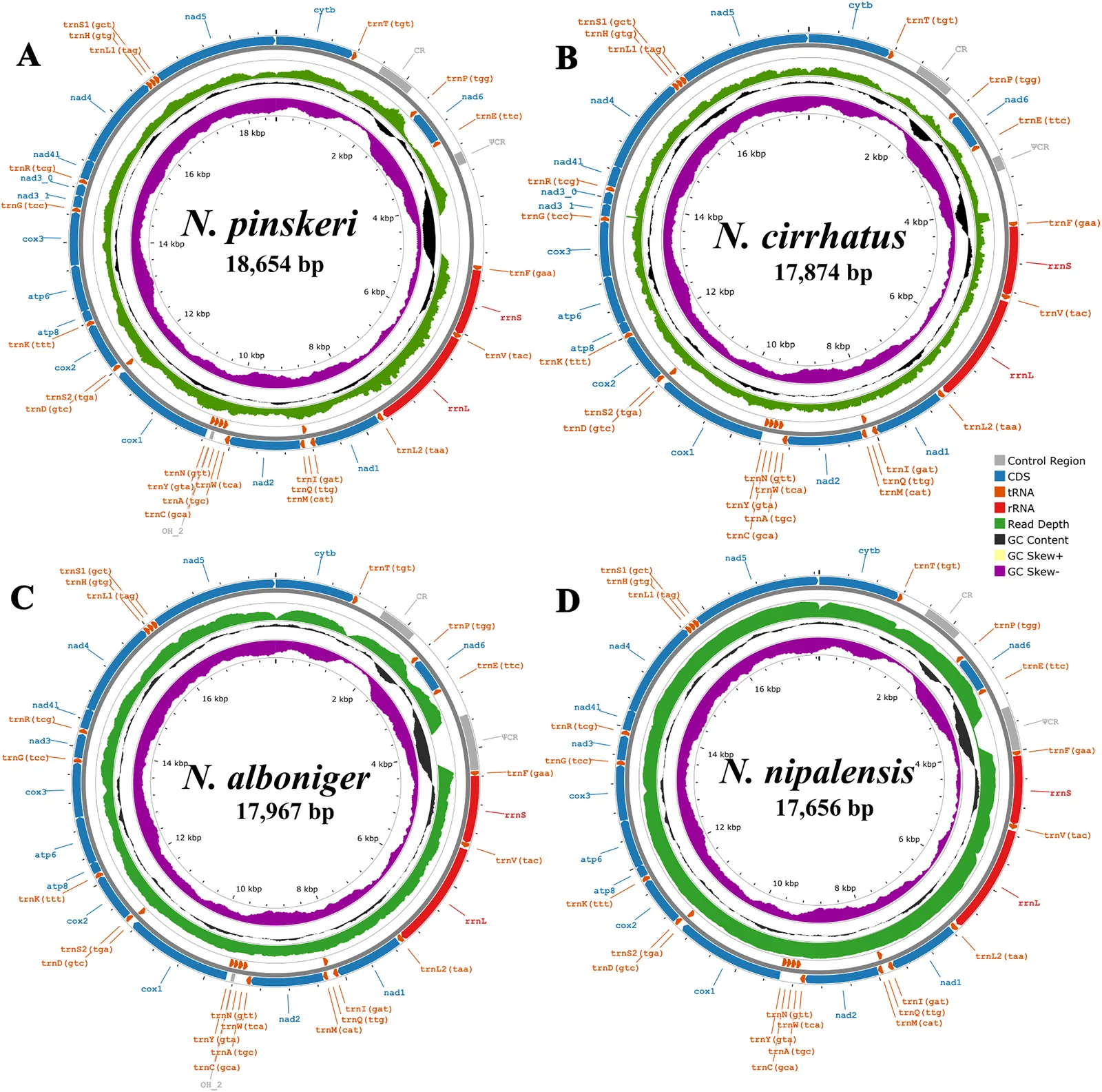
Annotated mitochondrial genomes of the genus *Nisaetus.* Graphical representation of features of assembled (A) *Nisaetus pinskeri* and (B) *Nisaetus cirrhatus* mitochondrial genomes generated by CGView in the Proksee web tool. Mitogenomes of (C) *Nisaetus nipalensis* (NC_007598.1) and (D) *Nisaetus alboniger* (NC_007599.1) were also re-annotated.

Meanwhile, the assembled mitochondrial genome of *N. cirrhatus* is 17,874 bp in length with a similar mitochondrial gene arrangement, shown in Fig 1B. Its length is much more in line with the other *Nisaetus* mitogenomes (Table 1). Both newly assembled mitogenomes are expected to be circular and double-stranded, with the heavy (positive) strand encoding the majority of the genes and the light (negative) strand only encoding the *nad6* and 8 tRNA genes (*trnP, trnE, trnQ, trnA, trnN, trnC, trnY, trnS2*). All tRNA genes were independently verified in an independent annotation with tRNAscan-SE.

Mapping the sequencing reads back to the assembled mitogenomes showed an average coverage of 154.91× for *N. pinskeri* (minimum = 25×) and 318.64× for *N. cirrhatus* (minimum = 27×). Read coverage was maintained across the manually joined contig boundary and the duplicated control-region-associated repeat region in both species (S2B and S2D Fig). Coverage across the overlap used for manual concatenation was approximately 100× in *N. pinskeri* and 500× in *N. cirrhatus*. The presence of mapped reads spanning this region is consistent with the assembled sequence. However, the exact repeat organization cannot be resolved confidently using short-read data alone, and future long-read sequencing could help confirm the structure of this region. Nucleotide content analysis reveals a prominent AT bias in the control regions, whereas the rest of the mitogenome has a slight GC bias. Nucleotide skew was measured similarly, and we observed all *Nisaetus* mitogenomes to have an overall negative GC skew, meaning the positive strand has more cytosines (C) than guanines (G).

Direct comparisons of nucleotide composition and skew of the four *Nisaetus* mitogenomes were represented by heatmaps in Fig 2A and 2B. The first nucleotide position of the protein-coding gene codons had the highest GC content of the three codon positions, averaging at 50% for all four mitogenomes. The tRNAs had the lowest GC content out of all gene regions. Nucleotide bias (AT- and GC-skew) was consistent throughout the genus, with the assembled mitogenomes showing a similar skew to already published mitogenomes. The *nad6* gene had the opposite skew from the rest of the protein-coding genes since it is encoded in the light strand.

**Fig 2:**
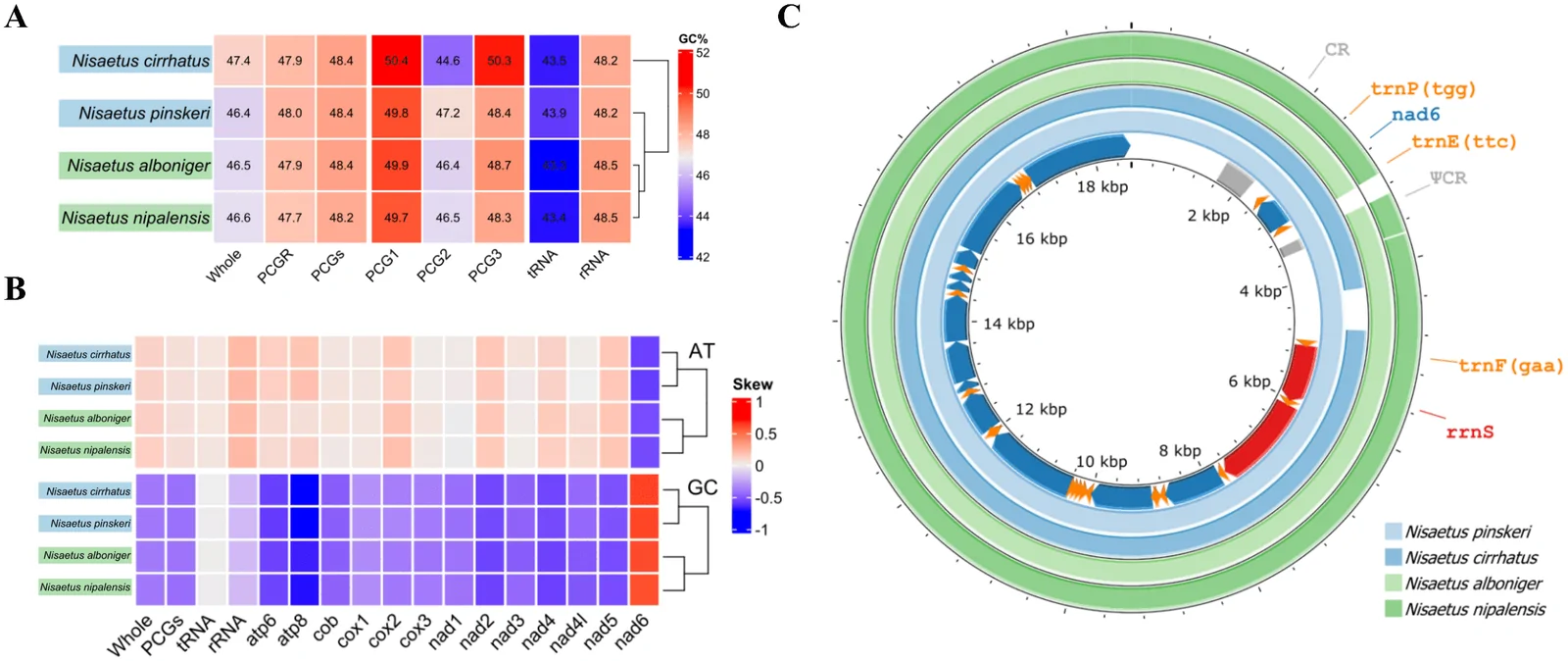
Summary of the mitogenome architecture of the genus *Nisaetus.* (A) Nucleotide (GC) content and skew of various gene regions in the mitogenome of the genus *Nisaetus* are shown in heatmaps. (B) Distribution map of represented *Nisaetus* hawk-eagles with shapefiles from the IUCN RedList. (C) BLAST alignment of *Nisaetus* mitogenomes with *Nisaetus pinskeri* as the backbone shows similarities in gene region arrangement.

The BLAST comparison of the *Nisaetus* mitogenomes using *N. pinskeri* as the reference showed that gene order and overall genome structure were highly conserved across the genus (Fig 2C). The primary difference was the expanded region between *trnE* and the pseudo-control region in *N. pinskeri*, corresponding to the duplicated control-region-associated repeat region. This difference is likely attributable to variation in the number of repeat units assembled in the control region rather than to large-scale structural differences among species. Because tandem repeats are difficult to resolve using short-read sequencing, the exact repeat copy number should be interpreted with caution and could be confirmed using long-read sequencing or targeted long-range PCR followed by Sanger sequencing [34].

### Comparative mitogenome analyses

The CR and pseudo-CR showed the highest sequence variability among the regions compared, while the rest of the mitogenome showed consistently high similarity across all taxa (Fig 3). Within the CR, *N. pinskeri* was most similar to Other *Nisaetus*, followed by Other Aquilinae, matching the monophyly of Aquilinae reported by [35]. Similarity to Other Accipitridae was lower. A single conserved block was observed between *trnT* and *trnP* across this group, with similarity scores decreasing toward more distantly related taxa.

**Fig 3:**
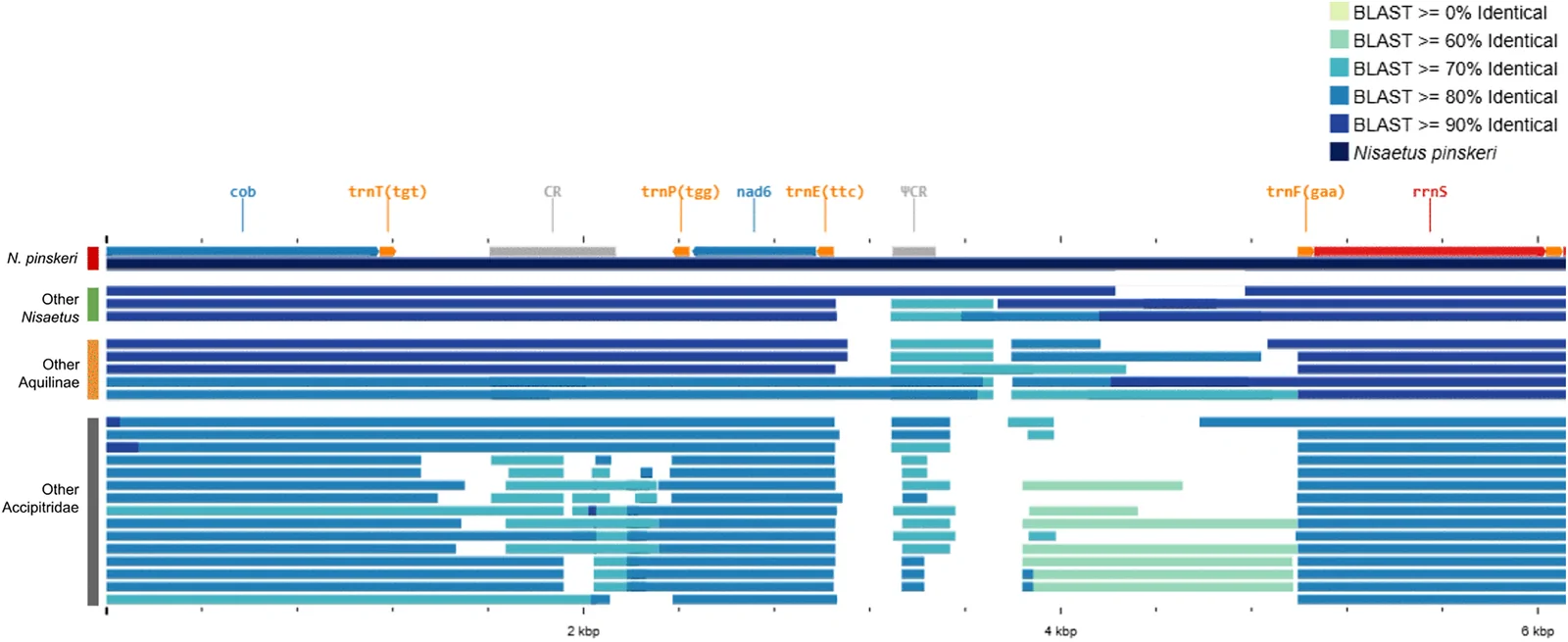
BLAST alignment of the control region (CR) and pseudo-control region (pseudo-CR) of *Nisaetus pinskeri* against representative Accipitridae mitogenomes. Sequences are grouped as: *Nisaetus pinskeri*; Other *Nisaetus* (*N. cirrhatus*, *N. nipalensis*, *N. alboniger*); Other Aquilinae (*Aquila chrysaetos*, *Aquila heliaca*, *Spizaetus tyrannus*, *Aquila nipalensis*, *Aquila fasciata*); and Other Accipitridae (*Spilornis cheela*, *Accipiter trivirgatus*, *Gyps fulvus*, *Accipiter virgatus*, *Circus melanoleucos*, *Buteo hemilasius*, *Accipiter gentilis*, *Haliaeetus albicilla*, *Buteo lagopus*, *Milvus migrans*, *Buteo buteo*, *Butastur liventer*, *Butastur indicus*, *Haliastur indus*, *Accipiter nisus*). Groups are separated by spacing for clarity. Mitochondrial genes flanking each region are labeled above the alignment.

In the pseudo-CR, extended conserved blocks were present in *Nisaetus* and in three Aquilinae taxa (*A. heliaca*, *A. nipalensis*, *A. fasciata*). Shorter conserved regions were observed in some Other Accipitridae taxa (Buteoninae). No conserved pseudo-CR sequence was recovered for Accipitrinae taxa in this alignment; these species have previously been reported to carry a duplicated control region [36].

Both control regions contain repeats near their 5’ and 3’ ends. Because repeats introduce ambiguity in short-read placement, the exact repeat number could not be resolved with confidence at the sequencing depth used here. These repeat regions are a plausible contributor to the approximately 1000 bp length difference observed between *N. pinskeri* and the other Nisaetus mitogenomes, though this cannot be confirmed without higher-coverage data.

The control region is widely used in phylogenetic and evolutionary studies due to its high variability; however, protein-coding genes may still be utilized when concatenated together for phylogenomics and genetic monitoring [37]. The protein-coding gene order of the newly assembled mitogenomes is identical to that of the mitogenomes of the genus *Nisaetus* and other raptors. The length of all protein-coding genes is 11,399 bp for *N. pinskeri* and 11,396 bp for *N. cirrhatus*. Most protein-coding genes are initiated with the start codon ATG, while the genes *cox1* and *nad3* are initiated with start codons GTG and ATC, respectively, similar to other *Nisaetus* raptors. In contrast, stop codons are more diverse, with codon TAA being the most common in all *Nisaetus* raptors. Other non-standard stop codons, CCT and AGA, were found in the *cox3* and *nad4* genes, respectively. The *nad1* genes of the assembled *N. cirrhatus* and *N. pinskeri* mitogenomes have an AGG stop codon, while *N. alboniger* and *N. nipalensis* have a TAA stop codon; both stop codons are present across the family Accipitridae.

The relative synonymous codon usage (RSCU) of the 30 publicly available mitogenomes of Accipitriformes and Cathartiformes species, with the 2 newly assembled *N. cirrhatus* and *N. pinskeri,* was visualized in a heatmap (Fig 4). These assembled mitogenomes show codon preferences similar to those of the other Accipitriformes and Cathartiformes mitogenomes examined. The codons ATG for methionine (Met) and TGG for tryptophan (Trp) are ubiquitously used across all mitogenomes. The three most used codons for both assembled mitogenomes were CUA (Leu), CUC (Leu), and AUC (Ile), while their least used codons vary widely, with GCG (Ala) and CGU (Arg) common in both.

**Fig 4:**
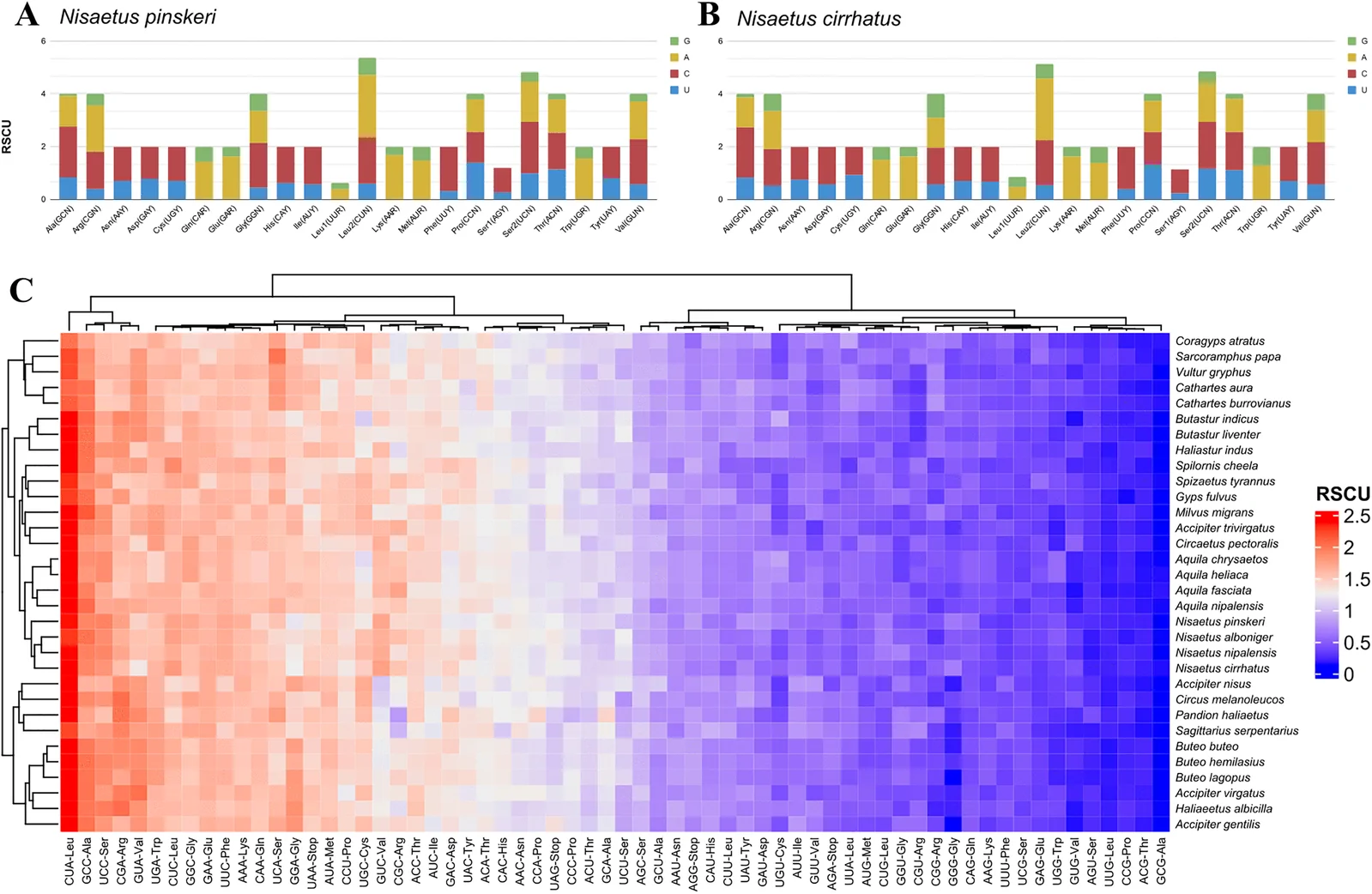
Relative synonymous codon usage (RSCU) of mitogenomes in the order Accipitriformes. RSCU values of the protein-coding region of (A) *Nisaetus pinskeri* and (B) *Nisaetus cirrhatus* per codon. (C) Heatmap of RSCU values of represented Accipitriformes mitogenomes.

RSCU values greater than 1 indicate positive codon usage bias, while values less indicate negative codon usage bias. Furthermore, RSCU values greater than 1.6 imply an overrepresentation of the codon, while values less than 0.6 imply an underrepresentation [38]. The codons with the highest RSCU values in assembled *N. cirrhatus* and *N. pinskeri* are CUA (Leu), UCC (Ser), and GCC (Ala), which are also the three codons with the highest average RSCU values of all mitogenomes studied here, indicating their prevalence throughout the family Accipitridae. The codon with the lowest RSCU value throughout all mitogenomes studied is GCG (Ala) with an average RSCU value of 0.12. Based on the specified criteria and average RSCU values, there are approximately 11 codons overrepresented and 22 underrepresented in the Accipitridae mitochondrial genome.

### Phylogenetic analysis

Phylogenetic studies are used in conservation biology to study evolutionary history and determine species units [39]. With the advent of new sequencing technologies, single-gene phylogenies are brought into question when compared to whole mitogenomic phylogenies that often have increased accuracy and statistical support [40]. Three datasets with a combination of mitochondrial protein-coding genes and RNAs of 27 Accipitriformes species and five Cathartiformes species were used for maximum likelihood tree construction under a partitioned framework, with model selection performed independently for each partition. *Cathartes aura* was selected as the outgroup based on its placement within Cathartiformes as the sister order to Accipitriformes [36].

The trees incorporating the protein-coding genes and all predicted genes provided similar topologies with consistent subfamily-level ordering and supported the monophyly of the family Accipitridae (Fig 5). Representatives of the order Cathartiformes formed a clade sister to Accipitriformes, though the ordinal status of Cathartidae remains debated; the IOC World Bird List does not recognize Cathartiformes as a separate order, instead placing Cathartidae within Accipitriformes [41,42].

**Fig 5:**
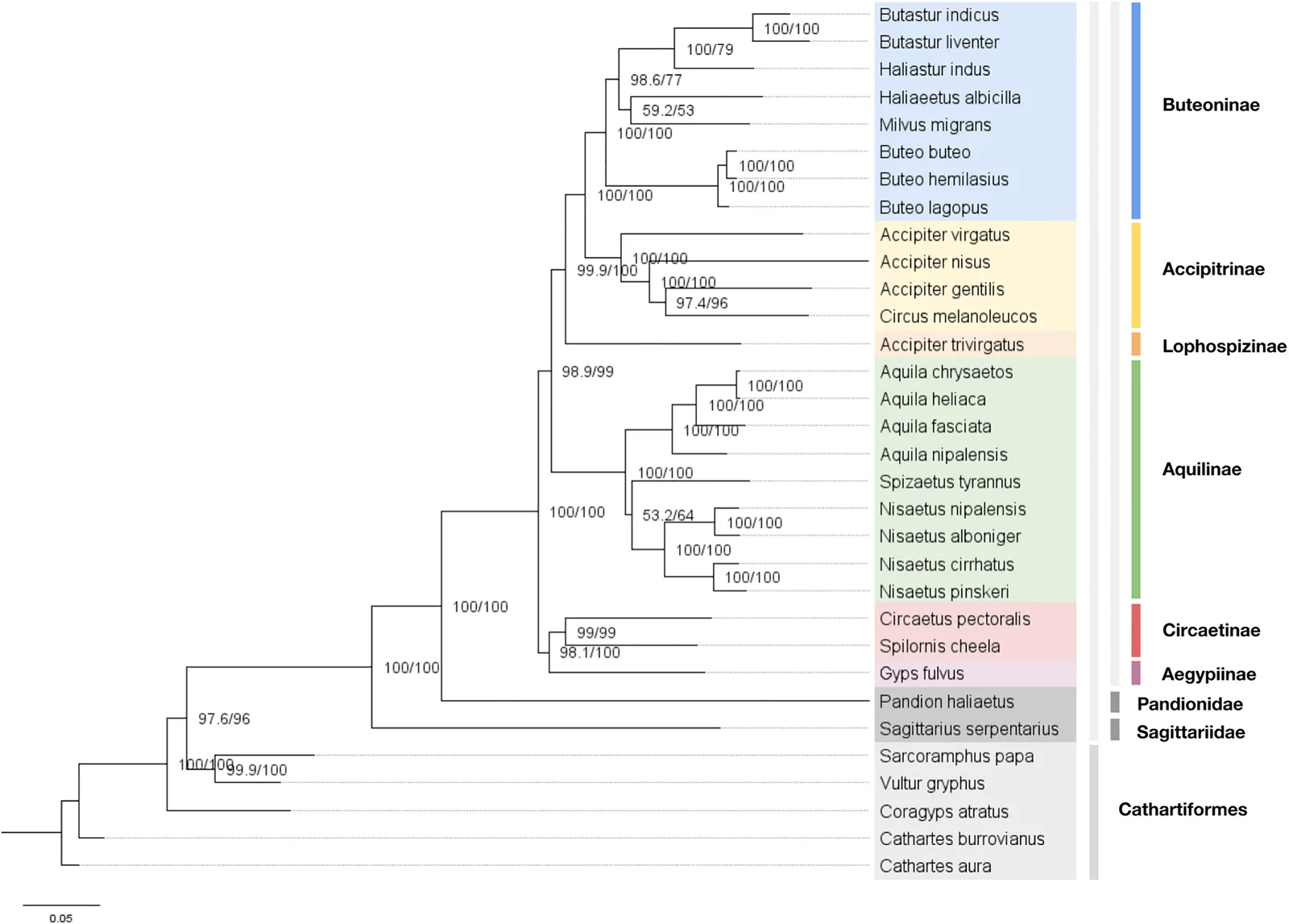
Maximum-likelihood phylogenetic tree of Accipitriformes inferred from a partitioned analysis of 12 protein-coding genes and 2 ribosomal RNA genes. *Cathartes aura* (Cathartiformes) was used as the outgroup. The dataset was partitioned by gene and codon position for protein-coding genes (first and second codon positions and third codon position as separate subsets) and by gene for ribosomal RNAs, with the best-fit substitution model selected independently for each partition using ModelFinder under the Bayesian Information Criterion. Node support values represent ultrafast bootstrap percentages (1000 replicates) and SH-aLRT values (1000 replicates), shown as SH-aLRT/UFBoot. Branches are colored by taxonomic group at the order, family, and subfamily levels.

Within Accipitriformes, the early-diverging lineages Pandionidae (*Pandion haliaetus*) and Sagittariidae (*Sagittarius serpentarius*) were recovered outside Accipitridae, consistent with their recognized familial distinctiveness [35]. Accipitridae comprised five clades corresponding to the represented subfamilies, along with *Accipiter trivirgatus*. The subfamilies Circaetinae and Aegypiinae formed a clade with Aquilinae in the multi-locus trees, which differs slightly from Catanach et al., who recovered Circaetinae and Aegypiinae as sister to the remaining Accipitridae [43]. Within Buteoninae, the subclades Milvini and Buteonini were not consistently recovered across datasets, as *Butastur* did not group with *Buteo* in the PCG or combined trees, though they were recovered together in the cox1 tree with moderate support (SH-aLRT/UFBoot = 84.9/77) [43].

In Accipitrinae, *Circus melanoleucos* was recovered within a non-monophyletic *Accipiter*, consistent with previous mitogenomic studies [44,45]. The positions of *Circus melanoleucos*, *Accipiter gentilis*, and *Accipiter nisus* varied across datasets, while *Accipiter virgatus* occupied a consistent position in the topology. This variability may reflect limited taxon sampling or the broader paraphyly of *Accipiter* as currently circumscribed. The IOC World Bird List now recognizes the split of *Accipiter* into multiple genera following Catanach et al., with *A. virgatus* reassigned to *Tachyspiza* and *A. gentilis* to *Astur* [41,43,46].

*Accipiter trivirgatus* was consistently recovered within the Buteoninae-Accipitrinae clade across all mitochondrial trees. This placement agrees with the findings of Catanach et al. and Sangster et al., both of which recommend its reclassification into the resurrected genus *Lophospiza* alongside *A. griseiceps* [43,47]. *Lophospiza* is now recognized by the IOC World Bird List [41].

Within Aquilinae, the genera *Aquila*, *Spizaetus*, and *Nisaetus* were represented. *Nisaetus* and *Spizaetus* were previously treated as a single genus before a mitochondrial phylogenetic study by Haring et al. separated New World hawk-eagles into *Spizaetus* and Southeast and East Asian hawk-eagles into *Nisaetus* [4]. Their morphological similarities, including prominent head crests, short, rounded wings, and robust talons, have been attributed to convergent evolution in response to similar tropical forest environments and prey [4]. Among the *Nisaetus* representatives, *N. pinskeri* and *N. cirrhatus* were recovered as more closely related to each other than to *N. alboniger* and *N. nipalensis*, a relationship also supported by the seven-locus dataset of Lerner et al. [48]. *N. cirrhatus* has the broadest distribution of the represented species, ranging from the Indian subcontinent to Southeast Asia, overlapping with all other *Nisaetus* taxa included here (Fig 2D).

*N. pinskeri* was once a subspecies of the Philippine Hawk-eagle (*Nisaetus philippensis*) but was treated as a separate species after a study by Gamauf et al. used *cytb* and CR to show the clear genetic distinction between the two. In the study, the split was hypothesized to have occurred earlier in the Pleistocene, when an ancestral population was split between the Greater Luzon and Greater Mindanao areas, which have been observed in other endemic bird groups in the Philippines [43,44]. Unfortunately, samples for *N. philippensis* were not obtained in this study to help prove this hypothesis.

*N. cirrhatus* comprises multiple subspecies, including ssp. *limnaeetus* in Southeast Asia and ssp. *ceylanensis* in Sri Lanka. Its former subspecies, *Nisaetus floris,* was elevated to species level based on morphological and genetic evidence [48,49]. During this study, a mitogenome assembly was attempted for *N. floris* using the raw reads used to assemble its draft genome (ASM2758013v1), but phylogenetic placement consistently recovered *Aquila fasciata* as its closest relative rather than any *Nisaetus* taxon. Investigation of the voucher specimen (MCZ Orn 261829, Museum of Comparative Zoology, Harvard University) suggests it was mislabeled, with the specimen likely representing *Aquila fasciata renschi*. If confirmed, the genome assembly ASM2758013v1 would require reannotation in the NCBI database.

Assessing the phylogenetic reliability of the constructed trees requires a comparative analysis against the latest Accipitridae tree reported by Catanach et al. [43]. The latest reported tree was pruned to only keep the species represented in this study for comparison with the constructed trees using the Generalized Robinson-Foulds metric (S10 Fig) [32]. The trees incorporating all protein-coding genes show the highest concordance with the reference phylogeny (GRF = 0.0584), while the 16S rRNA tree exhibited the greatest divergence (GRF = 0.1930), followed by the *cytb* tree (GRF = 0.1624), suggesting that these single-gene markers may be less reliable for recovering the overall phylogenetic relationships. The relatively poor performance of *cytb* has been previously attributed to the cumulative effects of parallel mutations and increased nucleotide sequence entropy in a study with the genus *Apodemus* by Lapinski et al [50]. Conversely, the *nad2* and *cox1* trees exhibit strong concordance with the latest reported tree, with GRF values of 0.0815 and 0.0836, respectively. This consistency suggests that these traditional gene markers may be more reliable for resolving phylogenetic relationships, particularly within Accipitriformes.

The Generalized Robinson-Foulds metric was calculated between the constructed trees. With respect to the all protein-coding gene tree, the *cox1* tree exhibited the highest concordance (GRF = 0.0255), followed by the combined PCG + RNA tree (GRF = 0.0561). Meanwhile, the greatest divergence among all reconstructed trees was observed between the *cox1* and 16S rRNA trees (GRF = 0.2083). Although the amino acid-based analysis was performed to evaluate whether the phylogenetic signal remained consistent after reducing the effects of synonymous substitutions and potential substitution saturation, the resulting tree (S4 Fig) showed lower concordance with the reference phylogeny (GRF = 0.1857) than the nucleotide-based PCG tree (GRF = 0.0584), indicating that nucleotide variation retains greater resolving power for the taxa examined in this study. These results highlight topological differences among single-gene phylogenies, suggesting that a multi-locus dataset may be required for accurately resolving taxonomic relationships. Accordingly, phylogenetic trees that incorporate all protein-coding genes show the highest concordance with the recently published, comprehensive phylogeny of the Accipitridae family by Catanach et al [43].

There is a cytosine (C) insertion at position 174 of the *nad3* gene that should cause a frameshift mutation in both *N. cirrhatus* and *N. pinskeri,* which has not been previously found in the other *Nisaetus* mitogenomes. This extra cytosine insertion is a reported phenomenon in raptors [13], owls [51], and other birds [52,53]. Proposed mechanisms for the functional translation despite the frameshift mutation include +1 frameshifting during translation and RNA editing [13].

The extra nucleotide is prevalent throughout the subfamilies of Accipitridae, albeit intermittently, as shown in Fig 6. Notably, the extra cytosine could be observed in the basal taxa of Accipitriformes (Pandionidae and Sagittariidae) and all Cathartiformes. The nucleotide is lost or gained in species of the same genus, such as in the case of *Aquila* and *Accipiter,* which could mean that the insertion has no phylogenetic significance, as inferred by similar studies [13]. However, it may be of evolutionary significance since all *nad3* frameshift events are limited to the Archosauria-Testudines group, providing evidence for the close relationship of turtles to birds and crocodiles [54].

**Fig 6:**
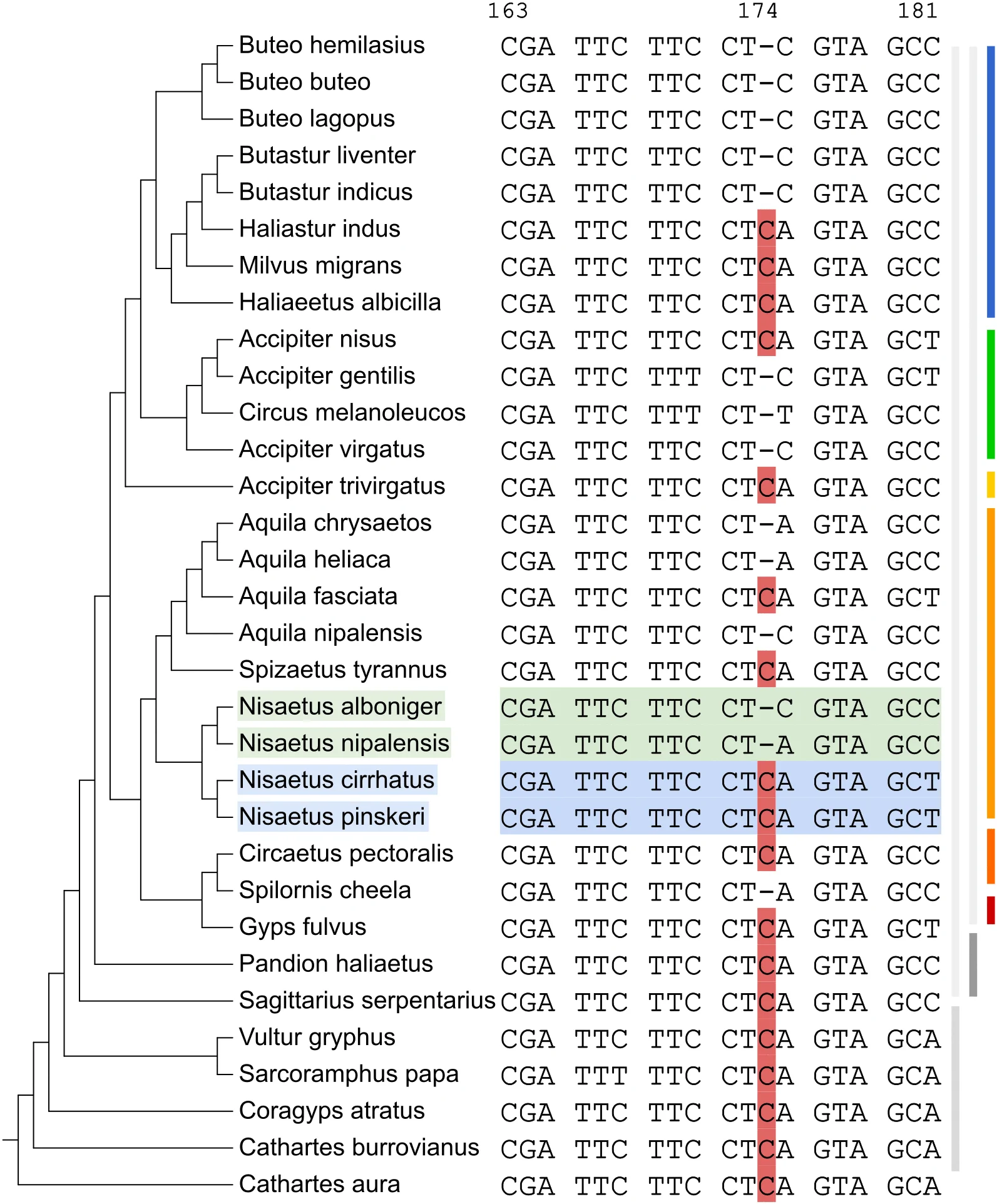
An extra cytosine (C) insertion at position 174 of the *nad3* gene appears intermittently throughout the order Accipitriformes. Cytosine insertion (red) was found in the assembled *N. cirrhatus* and *N. pinskeri* (blue) but not in the published *N. alboniger* and *N. nipalensis* (green) mitogenomes. A cladogram was modeled from the maximum likelihood tree based on all predicted genes.

### Evolutionary and diversity analyses

The variable sites and nucleotide diversity (pi) of the mitochondrial protein-coding genes of the 27 Accipitriformes raptors represented in this study were measured (Fig 7). The alignment of all 27 concatenated protein-coding gene sequences was 11,408 bp long, with 5,437 variable sites (47.7%). The *atp8* gene has the highest percentage of variable sites at 60.5%, while *cox1* has the lowest at 35.9%, consistent with a smaller-scale study with 11 mitochondrial sequences in the family Accipitridae [13]. The high percent variability of the *atp8* gene is also consistent with other birds from Passeriformes [55], and Strigiformes [51], and its short length (177 bp, 107 variable sites) is a plausible contributing factor.

**Fig 7:**
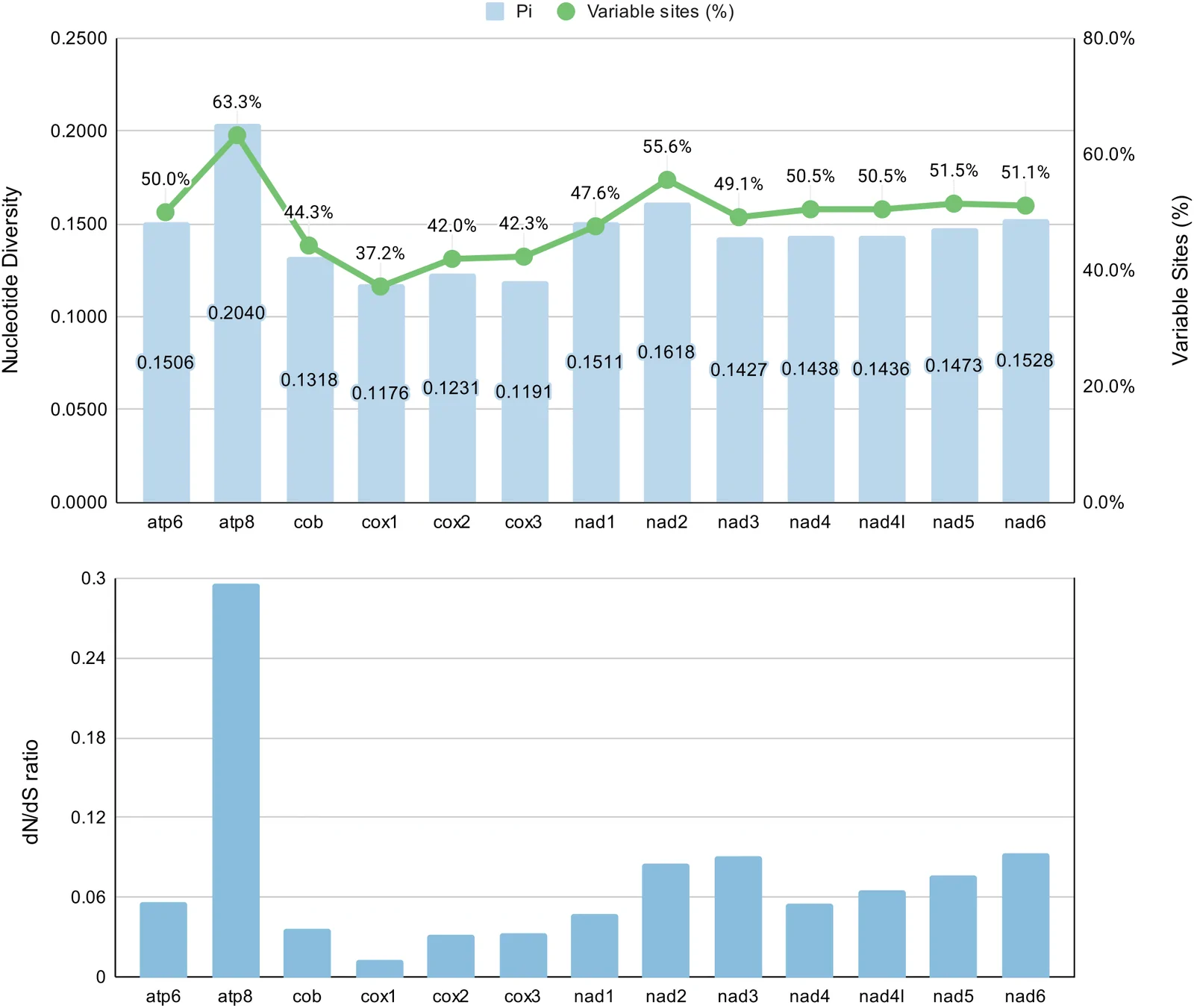
Nucleotide diversity and evolutionary analysis of each mitochondrial gene of order Accipitriformes. (Top) Nucleotide diversity of each gene was measured by pi and % variable sites. (Bottom) Evolutionary rates of each gene were estimated using the dN/dS ratio.

On the other hand, the *nad5* gene has the most variable sites with 890, but is also the longest at 1818 bp. The conserved sites of *cox1* are consistent with its established use as a mitochondrial barcode for species identification. The higher variability of *atp8* could be relevant for population genetic studies, though its short length may limit its utility [13].

The evolutionary rates of the mitochondrial protein-coding genes were measured using the ratio of non-synonymous to synonymous substitutions (dN/dS). All protein-coding genes had a dN/dS ratio of less than one, consistent with purifying selection acting on avian mitochondrial protein-coding genes generally. As with the nucleotide diversity analysis, *atp8* had the highest dN/dS ratio at 0.3040 and *cox1* the lowest at 0.0143, reflecting differences in the degree of purifying selection among genes rather than evidence of lineage-specific adaptive change.

## Conclusion

We report the assembly of complete mitogenomes for two *Nisaetus* species. The lengths of the mitogenomes of *N. pinskeri* and *N. cirrhatus* were 18,654 bp and 17,874 bp, with a GC content of 46.4% and 47.4%, respectively. Both mitogenomes consist of the typical avian mitochondrial genes (13 PCGs, 2 rRNAs, and 22 tRNAs) and expressed positive AT skew and negative GC skew in the heavy strand, while the opposite was observed for *nad6* in the light strand, similar to other *Nisaetus* mitogenomes. A cytosine insertion at position 174 of the *nad3* gene was observed for both assembled mitogenomes, which was not previously observed in other *Nisaetus* mitogenomes. BLAST analysis showed the presence of duplicated control regions prevalent in most Accipitriformes species. Mitogenomic analysis using all predicted genes supported the basal position of Cathartiformes, which is sister to Accipitriformes.

Furthermore, the results corroborated the position of the 5 subfamilies and the phylogenetic separation of *Accipiter trivirgatus* in the family Accipitridae. All mitochondrial protein-coding genes undergo purifying selection, with the highest dN/dS detected for *atp8* at 0.3040 and the lowest for *cox1* at 0.0143. These assembled mitogenomes provide reference sequences that may support future population genetic, phylogeographic, and conservation genomic studies of these species.

## Supporting information

S1 Fig Mitochondrial genomes generated by other organelle assemblers. Supplementary assemblies were generated using MitoZ (A), MitoFinder (B), and GetOrganelle (C). Assemblies from MitoZ and MitoFinder did not recover the *nad6-trnP-trnE* gene block located between the control region and pseudo-control region on the negative strand. GetOrganelle recovered all expected avian mitochondrial genes but assembled only three repeat units within the duplicated control-region-associated repeat region, compared to 24 repeat units recovered by NOVOPlasty.
(DOCX)

S2 Fig Repeat motif of control region and sequence depth plots of generated mitogenomes. (A) Repeat motifs found in the control regions of several *Nisaetus* species. (B) Sequence depth plot of *Nisaetus pinskeri* assembled by NOVOPlasty and manually concatenated, keeping 24 instances of the repeat motif (highlighted by a yellow box). (C) Sequence depth plot of *N. pinskeri* assembled by GetOrganelle, which only contains 3 repeat units (highlighted by a yellow box). (D) Sequence depth plot of *N. cirrhatus* assembled by NOVOPlasty with repeat units highlighted by a yellow box.
(DOCX)

S3 Fig Maximum-likelihood tree of Accipitriformes based on all mitochondrial protein-coding genes (nucleotide). (DOCX)

S4 Fig Maximum-likelihood tree of Accipitriformes based on all mitochondrial protein-coding genes (amino acid). (DOCX)

S5 Fig Maximum-likelihood tree of Accipitriformes based on all mitochondrial ribosomal RNAs. (DOCX)

S6 Fig Maximum-likelihood tree of Accipitriformes based on the *cytb* gene. (DOCX)

S7 Fig Maximum-likelihood tree of Accipitriformes based on the *cox1* gene. (DOCX)

S8 Fig Maximum-likelihood tree of Accipitriformes based on the *nad2* gene. (DOCX)

S9 Fig Maximum-likelihood tree of Accipitriformes based on the 16S rRNA. (DOCX)

S10 Fig Pairwise Generalized Robinson-Foulds distances between each constructed Maximum-Likelihood tree. (DOCX)

S1 Table Mitogenome annotation of *Nisaetus pinskeri.* Complete annotation of partial mitochondrial genome (18,654 bp) including protein-coding genes, ribosomal RNAs, transfer RNAs, and putative origin of replication regions. Gene features are listed with start and stop positions, strand orientation, lengths, intragenic distances, anticodons, and start/stop codons where applicable.
(XLSX)

S2 Table Mitogenome annotation of *Nisaetus cirrhatus.* Complete annotation of partial mitochondrial genome (17,874 bp) including protein-coding genes, ribosomal RNAs, transfer RNAs, and putative origin of replication regions. Gene features are listed with start and stop positions, strand orientation, lengths, intragenic distances, anticodons, and start/stop codons where applicable.
(XLSX)
