# Supplementary material for: The mitochondrial genomes of the endemic and endangered South Philippine Hawk-eagle Nisaetus pinskeri and other Nisaetus Hawk-eagles (Aves, Accipitriformes)
Source: PLoS One. 2026 Oct 26;21(10):e0360538. doi: 10.1371/journal.pone.0360538 (PMC13704876; doi:10.1371/journal.pone.0360538)
Supplement: Supplementary file 1 [file pone.0360538.s001.docx]

**S1 Figure. Mitochondrial genomes generated by other organelle assemblers.** Supplementary assemblies were generated using MitoZ (A), MitoFinder (B), and GetOrganelle (C). Assemblies from MitoZ and MitoFinder did not recover the *nad6-trnP-trnE* gene block located between the control region and pseudo-control region on the negative strand. GetOrganelle recovered all expected avian mitochondrial genes but assembled only three repeat units within the duplicated control-region-associated repeat region, compared to 24 repeat units recovered by NOVOPlasty.
