# Supplementary material for: The mitochondrial genomes of the endemic and endangered South Philippine Hawk-eagle Nisaetus pinskeri and other Nisaetus Hawk-eagles (Aves, Accipitriformes)
Source: PLoS One. 2026 Oct 26;21(10):e0360538. doi: 10.1371/journal.pone.0360538 (PMC13704876; doi:10.1371/journal.pone.0360538)
Supplement: Supplementary file 2 [file pone.0360538.s002.docx]

**S2 Figure. Repeat motif of control region and sequence depth plots of generated mitogenomes.** (A) Repeat motifs found in the control regions of several *Nisaetus* species. (B) Sequence depth plot of *Nisaetus pinskeri* assembled by NOVOPlasty and manually concatenated, keeping 24 instances of the repeat motif (highlighted by a yellow box). (C) Sequence depth plot of *N. pinskeri* assembled by GetOrganelle, which only contains 3 repeat units (highlighted by a yellow box). (D) Sequence depth plot of *N. cirrhatus* assembled by NOVOPlasty with repeat units highlighted by a yellow box.
