# Supplementary material for: The mitochondrial genomes of the endemic and endangered South Philippine Hawk-eagle Nisaetus pinskeri and other Nisaetus Hawk-eagles (Aves, Accipitriformes)
Source: PLoS One. 2026 Oct 26;21(10):e0360538. doi: 10.1371/journal.pone.0360538 (PMC13704876; doi:10.1371/journal.pone.0360538)
Supplement: Supplementary file 4 [file pone.0360538.s004.docx]

**S4 Figure. Maximum-likelihood tree of Accipitriformes based on all mitochondrial protein-coding genes (amino acid).**
