# Supplementary material for: The mitochondrial genomes of the endemic and endangered South Philippine Hawk-eagle Nisaetus pinskeri and other Nisaetus Hawk-eagles (Aves, Accipitriformes)
Source: PLoS One. 2026 Oct 26;21(10):e0360538. doi: 10.1371/journal.pone.0360538 (PMC13704876; doi:10.1371/journal.pone.0360538)
Supplement: Supplementary file 11 [file pone.0360538.s013.docx]

This manuscript addresses an important conservation-relevant topic and provides useful mitochondrial genomic data for *Nisaetus* hawk-eagles, especially for the endangered *Nisaetus* *pinskeri*. However, the manuscript would benefit from revision because several textual inconsistencies, methodological ambiguities, and interpretive overstatements need to be addressed

Comments:

- The manuscript should state much more clearly which mitogenomes were newly sequenced by the authors and which were assembled from publicly available reads. In the Methods, *N. pinskeri* was sequenced from newly collected blood samples, whereas *N. cirrhatus* and *N. floris* read datasets were downloaded from public archives, so the wording in the title, abstract, and conclusion should be aligned with that distinction.
- The manuscript reports the length of *N. cirrhatus* as 17,873 bp in the abstract and conclusion, but 17,874 bp in the Results and in Figure 1. Please reconcile this discrepancy throughout the manuscript
- In the Results, the *N. pinskeri* mitogenome is described as containing 13 PCGs, 20 tRNAs, 2 rRNAs, one control region, and one pseudo-control region, whereas the Conclusion states that both mitogenomes consist of the typical avian set of 13 PCGs, 2 rRNAs, and 22 tRNAs. Please clarify the final annotated gene content and ensure that the Results, figures, and Conclusion are fully consistent.
- The phylogenetic Methods section states that ‘the protein model’ with the lowest AIC/BIC was chosen, but the trees were then inferred using nucleotide substitution models such as GTR+G+I and TN93+G+I. Please clarify whether the analyses were performed on nucleotide alignments, amino-acid alignments, or both, and revise the model-selection description accordingly.
- The maximum-likelihood analyses were run with 100 bootstrap replicates only, which is relatively modest for a study making several topological comparisons across datasets.
- Table 1 appears to contain a classification problem because *Circaetus pectoralis* is listed under ‘Cathartidae,’ which is inconsistent with the broader structure of the table and with the manuscript’s own discussion of Circaetinae.

Minor comments:

- Please replace ‘singular mitochondrial genes’ with ‘single mitochondrial genes,’ which is the more natural term in this context
