# Supplementary material for: The mitochondrial genomes of the endemic and endangered South Philippine Hawk-eagle Nisaetus pinskeri and other Nisaetus Hawk-eagles (Aves, Accipitriformes)
Source: PLoS One. 2026 Oct 26;21(10):e0360538. doi: 10.1371/journal.pone.0360538 (PMC13704876; doi:10.1371/journal.pone.0360538)
Supplement: Supplementary file 12 [file pone.0360538.s015.docx]

### **Response to the Academic Editor**

**Editor Comment:** "As you will see from these reports, the reviewers do not have many concerns about the novelty and value of your data and only very limited concerns about the way you have analyzed your data. Most concerns are related to a perceived over interpretation of your data. In my view this can be dealt with by reducing the length of your discussion and restricting your speculation about the significance of your findings (focusing specifically on what you have found without much extrapolation on what that might mean)."

**Response:** We thank the Editor for this guidance. We have substantially revised the Results, Discussion, and Conclusion to focus on describing our findings directly, removing speculative extrapolation regarding adaptive significance and conservation implications not directly supported by our data.

**Editor Comment:** "Two of your reviewers specifically raised concerns about the claims that your mitogenomes are complete. For your work to be published in PLOS ONE you must address this concern by either: (1) changing how your refer to your mitogenomes (i.e as draft genomes, or near-complete genomes et cetera) or (2) by providing additional supporting data justifying your complete genome claim or (3) by rebutting the reviewers´ criticisms with clear reasoned justifications for your use of the term "complete" mitogenomes."

**Response:** We have switched to "near-complete" or "draft" mitogenome when referring to both the *Nisaetus pinskeri* and *Nisaetus cirrhatus* throughout the whole paper.

### **Response to Reviewer #1**

**Reviewer #1:** "This manuscript reports a new mitochondrial genome from an interesting and endangered bird species. The sequencing, assembly, and downstream analyses appear technically sound, detailed, and follow established standards. I have no major methodological concerns, except that some analyses are interesting, but might be an overkill (FIG 3 and 4)."

**Response:** We thank the reviewer for this positive assessment of our methodology. We have revised Figure 3 to improve its interpretability. We have retained Figure 4 (RSCU analysis) as codon usage bias is a standard component of mitogenome resource papers and provides useful reference data for future comparative studies, but have shortened the accompanying text to a more descriptive treatment.

### **Response to Reviewer #2**

**Reviewer #2:** "The manuscript reports the mitochondrial genomes of the southern Philippine endemic and endangered Nisaetus pinskeri and Nisaetus cirrhatus, and compares them with mitochondrial genomes of other Accipitriformes birds. The study has certain conservation biological significance. Therefore, this study has some resource value and publication potential. However, in its current form, the manuscript still has several important issues, especially regarding the reliability of mitochondrial genome assembly, the methodological rigor of the phylogenetic analyses, whether the results sufficiently support the conclusions, and whether the conservation implications are overstated."

**Response:** We thank the reviewer for this assessment and address each specific issue raised below.

**Reviewer #2:** "The most serious issue is that the authors clearly state that the mitochondrial genome of N. pinskeri was obtained by manually concatenating two contigs, while also noting that the control region and pseudo-control region have low coverage and contain repeat-rich regions. In the absence of evidence from long-range PCR, Sanger sequencing, long-read sequencing, junction-spanning reads, or assembly graphs, the current evidence is insufficient to support the claim that it represents a "complete mitochondrial genome". Therefore, the authors should either provide additional validation evidence or describe this mitogenome more cautiously throughout the manuscript as "near-complete", "tentative", or "putatively assembled"."

**Response:** We have switched to "near-complete" or "draft" mitogenome when referring to both the *Nisaetus pinskeri* and *Nisaetus cirrhatus* throughout the whole paper.

**Reviewer #2:** "The authors do not provide a sufficiently comprehensive review of existing molecular phylogenetic studies on the genus Nisaetus. In particular, the taxonomic history of N. pinskeri, which was previously treated as a subspecies of N. philippensis, and the support from existing cytb and control-region sequences for distinguishing these taxa should be more clearly described in the Introduction."

**Response:** We have added this part: “Likewise, *N. pinskeri* was formerly considered a subspecies of the Philippine hawk-eagle (*N. philippensis*) but was elevated to species status following a mitochondrial study by Gamauf et al., also using cytb and control region sequences, that demonstrated clear genetic differentiation, likely resulting from an early Pleistocene geographic isolation between the Greater Luzon and Greater Mindanao faunal regions” in the Introduction.

**Reviewer #2:** "Second, although the authors emphasize that complete mitochondrial genomes provide higher resolution than single-gene markers, they do not adequately discuss the inherent limitations of mitochondrial data, such as maternal inheritance, representation of a single genetic background, lack of recombination, and possible discordance with nuclear genomic phylogenies."

**Response:** We have added this part: “Nevertheless, the maternal inheritance and lack of recombination of mtDNA mean that mitogenomes represent a single evolutionary history, which may not always reflect species relationships.” in the Introduction.

**Reviewer #2:** "Third, the conservation biological significance is stated rather broadly. One or a few mitochondrial genomes can provide useful reference sequences, but they cannot directly reveal population genetic diversity, levels of inbreeding, effective population size, or conservation management units."

**Response:** We have revised the Discussion and Conclusion to state that these mitogenomes serve as reference sequences for future population genetic, phylogeographic, and conservation genomic studies, rather than as direct sources of conservation-management conclusions.

**Reviewer:** "The authors should at least provide the following information: the number of raw reads, the number of filtered reads, the Q30 percentage, the average mitochondrial coverage, the minimum coverage, contig lengths, the repeat-sequence features at the contig ends, the specific basis for manual concatenation, and whether junction-spanning reads support the inferred connection."

**Response:** We added “Quality control using Fastp yielded a total of 65.09 G raw reads, of which 64.77 G remained after filtering, with a Q30 percentage of 92.96%, providing the basis for subsequent mitochondrial assembly and coverage analyses.” at the start of the results. We also added S2 Figure to show the repeat-sequence features at the contig ends and the sequence depth plot.

**Reviewer #2:** "In addition, the authors are encouraged to use other mitochondrial genome assembly programs, such as MitoZ, GetOrganelle, or MitoFinder, for cross-validation. If no experimental validation or additional assembly evidence can be provided, the term "complete mitogenome" should be avoided."

**Response:** We have performed the additional analyses using the three suggested assemblers and have provided the output in S1 Figure. We have deemed that the NOVOPlasty output still has the most merit. We have avoided the term “complete mitogenome” throughout the revised manuscript.

**Reviewer #2:** "Regarding the selection pressure analysis, the authors used SLAC to calculate Ka/Ks, but the methodological description is insufficient. They should clarify whether the input files were codon alignments, whether each gene was analyzed separately, whether a phylogenetic tree was used, and whether stop codons and anomalous insertion sites were removed."

**Response:** We have added “which requires codon-based multiple sequence alignments and a corresponding phylogenetic tree as input (see Mitogenomic phylogenetic analysis)” to the methodology to clarify the input files. Stop codons and insertions were removed.

**Reviewer #2:** "The Discussion is somewhat unfocused. Some sections, such as the reclassification of the genus Accipiter, the taxonomic controversy between Cathartiformes and Accipitriformes, and the possible mislabeling of the Nisaetus floris sample, are interesting but not closely connected to the core contribution of this manuscript. These side discussions should be condensed, and the Discussion should focus more clearly on the two newly assembled mitochondrial genomes, the uncertainty in control-region structure, comparisons within Nisaetus, and the value of these data for future research."

**Response:** We have condensed the sections on Accipiter reclassification and the Cathartiformes/Accipitriformes taxonomic controversy to brief, single-sentence mentions with citation, and refocused the Discussion on the two newly assembled genomes, control-region structural uncertainty, within-Nisaetus comparisons, and future research value, per the reviewer's suggestion.

### **Response to Reviewer #3**

**Reviewer #3:** "This manuscript reports mitogenomic data for Nisaetus pinskeri and Nisaetus cirrhatus and provides comparative analyses with other Accipitriformes/Cathartiformes mitogenomes... However, the current manuscript is closer to a mitogenomic resource report with routine comparative analyses, and several key claims are not sufficiently supported."

**Response:** We thank the reviewer for this assessment and address the specific concerns below.

**Reviewer #3:** "The most serious issue is that the N. pinskeri mitogenome was assembled as two contigs and then manually concatenated, but the manuscript does not provide sufficient technical details to explain how this manual joining was performed... Therefore, the claim of a "complete mitogenome" requires additional validation or more cautious wording."

**Response:** We have switched to "near-complete" or "draft" mitogenome when referring to both the *Nisaetus pinskeri* and *Nisaetus cirrhatus* throughout the whole paper.

**Reviewer #3:** "Second, the phylogenetic analyses also require substantial revision. The manuscript appears to use only 100 bootstrap replicates, which is insufficient... the basis for choosing the reported models, including GTR+G+I and TN93+G+I, is not clearly justified. These models should be selected using an appropriate model-selection procedure, such as ModelFinder or a comparable tool... Without a more rigorous phylogenetic framework, including appropriate model selection, partitioned analyses, and at least 1000 bootstrap or ultrafast bootstrap replicates, the resulting topology and the evolutionary interpretations based on it are not sufficiently reliable."

**Response:** The original trees were reconstructed using IQ-TREE2 with a fully partitioned analysis framework. For the protein-coding gene (PCG) datasets, each gene was partitioned by codon position into first and second codon positions (pos12) and third codon position (pos3) as separate subsets. Ribosomal RNA genes (rrnS and rrnL) were each treated as a single partition. Model selection was performed independently for each partition using ModelFinder implemented in IQ-TREE2, with the best-fit substitution model selected under the Bayesian Information Criterion (BIC). Partition schemes were further optimized by merging subsets with statistically indistinguishable models using the greedy merging algorithm in ModelFinder, reducing the risk of overparameterization. The GTR+G+I and TN93+G+I models used in the original manuscript were replaced by partition-specific models selected through this procedure, and are now reported in full in the manuscript. Node support was assessed using 1000 ultrafast bootstrap replicates (UFBoot2) and 1000 SH-aLRT replicates.

To address the reviewer's concern regarding amino acid datasets, each PCG alignment was also translated using the vertebrate mitochondrial genetic code (NCBI translation table 2) and analyzed as a separate amino acid dataset, with model selection restricted to mitochondrial substitution matrices (mtREV, mtZOA, mtMAM, mtART) alongside general protein models (WAG and LG). The amino acid-based topology was consistent with the nucleotide-based partitioned trees, supporting the robustness of the inferred relationships.

The revised methods, updated model assignments per partition, and revised phylogenetic trees are reflected in the updated manuscript.

**Reviewer #3:** "The claim of a "complete mitogenome" for N. pinskeri is not sufficiently supported... The authors should provide additional evidence supporting the manually joined assembly, especially around the contig junctions, control region, pseudo-control region, and repeat-rich regions. Ideally, the assembly should be validated using long-range PCR/Sanger sequencing or long-read sequencing. If such validation cannot be provided, the authors should avoid repeatedly using "complete mitogenome" or "complete mitochondrial genome" and instead use more cautious wording... All conclusions based on genome length, repeat number, and control-region structure should also be weakened accordingly."

**Response:** We have switched to "near-complete" or "draft" mitogenome when referring to both the *Nisaetus pinskeri* and *Nisaetus cirrhatus* throughout the whole paper.

**Reviewer #3:** "Because the control region and pseudo-control region have low coverage and repetitive sequences, the authors should test additional mitogenome assembly tools, such as MitoZ, MitoFinder, GetOrganelle... The authors should also provide sequencing-depth plots across the entire mitogenome... The statement that read depth was estimated using MitoZ is not sufficiently transparent. Although MitoZ may provide coverage-related information, the authors should map the raw reads back to the assembled mitogenome using a standard approach, such as BWA, Bowtie2, or minimap2, and calculate coverage using SAMtools depth, SAMtools coverage, or bedtools genomecov."

**Response:** We have performed the additional analyses using the three suggested assemblers and have provided the output in S1 Figure. We have deemed that the NOVOPlasty output still has the most merit. We also added S2 Figure to show the repeat-sequence features at the contig ends and the sequence depth plots.

**Reviewer #3:** "It is unclear whether the tRNA annotations inferred by tRNAscan-SE were fully consistent with those generated by MITOS2. Inconsistencies between these two tools are common in mitochondrial genome annotation, and the authors should briefly explain how the final tRNA annotations were determined."

**Response:** All tRNA genes were independently verified in an independent annotation with tRNAscan-SE.

**Reviewer #3:** "For mitochondrial protein-coding genes, the authors should use an amino-acid-guided or codon-aware alignment strategy... Ribosomal RNA genes and tRNA genes should be aligned separately using methods appropriate for RNA sequences. The authors should clearly describe how each class of genes was aligned, trimmed, and concatenated."

**Response:** We have revised the alignment strategy accordingly. Protein-coding genes were aligned using MACSE v2, which performs codon-aware alignment directly on nucleotide sequences while accounting for frameshifts, using the vertebrate mitochondrial genetic code. This approach preserves codon structure throughout the alignment and is equivalent to amino acid-guided alignment in its treatment of reading frame integrity. Frameshift characters introduced by MACSE were replaced with standard gap characters prior to downstream analyses. Ribosomal RNA genes (rrnS and rrnL) were aligned separately using MUSCLE with default settings, as this approach is appropriate for structural RNA sequences that lack codon structure. All alignments were trimmed using trimAl with the '-automated1' setting, which is optimized for maximum likelihood analyses. The trimmed alignments were then concatenated into the three multi-locus datasets described in the Methods.

**Reviewer #3:** "A mitochondrial genome represents a single maternally inherited linkage unit and cannot by itself represent the species tree. The authors should avoid overinterpreting the mitochondrial topology, especially for higher-level relationships within Accipitriformes. The phrase "basal taxa of Accipitriformes" should also be avoided... A more appropriate wording would be "early-diverging lineages within Accipitriformes, including Pandionidae and Sagittariidae.""

**Response:** We have replaced "basal" with "early-diverging" throughout the manuscript when referring to extant lineages (e.g., "early-diverging lineages within Accipitriformes, including Pandionidae and Sagittariidae," per the reviewer's suggested phrasing), and have added a caveat noting that the mitochondrial phylogeny reflects a single maternally inherited locus.

**Reviewer #3:** "The reported extra cytosine at position 174 of the nad3 gene is interesting. However, the authors should clearly explain how this insertion was handled during translation, sequence alignment, and dN/dS analysis. Since this insertion may cause an apparent frameshift but is known in birds and may involve translational frameshifting or RNA editing, it is important to clarify whether the insertion was retained, removed, or manually corrected in each downstream analysis."

**Response:** The extra cytosine at position 174 was manually corrected in each downstream analysis.

**Reviewer #3:** "The authors used SLAC/Datamonkey to calculate Ka/Ks or dN/dS values and found that all mitochondrial PCGs are under purifying selection... A single overall dN/dS value per gene across Accipitridae or Accipitriformes only indicates general conservation; it does not support claims regarding environmental adaptation... Otherwise, this section should be presented as a descriptive comparison of mitochondrial gene conservation."

**Response:** We have revised this section to be more descriptive and less speculative regarding their involvement in environmental adaptation.

**Reviewer #3:** "The manuscript repeatedly suggests that these mitogenomes can provide critical insights for genetic diversity monitoring and conservation management... The authors should state more cautiously that these mitogenomes provide reference sequences for future population genetic, phylogeographic, and conservation genomic studies."

**Response:** We have already revised the Conclusion and relevant Discussion passages which now state that the assembled mitogenomes "provide reference sequences that may support future population genetic, phylogeographic, and conservation genomic studies," and the closing sentence advocating direct conservation-strategy application has been removed.

**Reviewer #3:** "Fig. 3 is difficult to interpret in its current form... The authors should either redesign Fig. 3 with clearer ring labels and a more informative legend, or consider replacing it with a more interpretable visualization method."

**Response:** We have redesigned Figure 3 to focus on the CR/pseudo-CR specifically, with taxa grouped into four labeled categories (*N. pinskeri*, Other *Nisaetus*, Other Aquilinae, Other Accipitridae), spaced groupings, and an expanded caption.

#### **Minor Comments**

**Reviewer #3:** "The abstract should avoid overly strong phrases such as "critical insights" unless the conclusions are directly supported by the analyses."
**Response:** We have revised the abstract to remove "critical insights" and similar overstated phrasing, consistent with the Discussion/Conclusion revisions described above.

**Reviewer #3:** "Please ensure consistency in the reported length of the N. cirrhatus mitogenome. The abstract reports 17,873 bp, whereas the results section reports 17,874 bp."
**Response:** We have corrected this and report 17,874 bp for the *N. cirrhatus* mitogenome

**Reviewer #3:** "The phrase "family Accipitriformes" should be corrected, as Accipitriformes is an order, not a family."
**Response:** Corrected to "family Accipitridae”

**Reviewer #3:** ""itsadaptability" appears to lack a space."
**Response:** Corrected to "its adaptability."

**Reviewer #3:** "The attempted assembly of the Nisaetus floris dataset and its subsequent identification as likely Aquila fasciata are potentially important, but this part is currently unclear. The authors should clarify whether this re-identification is part of the present study and, if so, what evidence supports it... The authors should also state whether the N. floris dataset was excluded from all downstream analyses and whether this potential misidentification has been reported to the relevant database or museum collection."

**Response:** During mitogenome assembly of the dataset associated with the draft genome ASM2758013v1, the resulting sequence was consistently recovered as sister to *Aquila fasciata* rather than to any *Nisaetus* taxon. We then examined the associated BioSample metadata and voucher specimen (MCZ Orn 261829, Museum of Comparative Zoology, Harvard University), which has a documented history of being reclassified from *Nisaetus floris* to *Aquila fasciata*. Based on this evidence, we concluded that the specimen was likely mislabeled. We have also clarified that the ASM2758013v1 dataset was excluded from all downstream phylogenetic analyses presented in this study. Finally, we now explicitly state that the potential misidentification has not yet been formally reported to the NCBI.

### **Response to Reviewer #4**

**Reviewer #4:** "The manuscript reports the length of N. cirrhatus as 17,873 bp in the abstract and conclusion, but 17,874 bp in the Results and in Figure 1. Please reconcile this discrepancy throughout the manuscript."

**Response:** We have corrected this and report 17,874 bp for the *N. cirrhatus* mitogenome

**Reviewer #4:** "In the Results, the N. pinskeri mitogenome is described as containing 13 PCGs, 20 tRNAs, 2 rRNAs, one control region, and one pseudo-control region, whereas the Conclusion states that both mitogenomes consist of the typical avian set of 13 PCGs, 2 rRNAs, and 22 tRNAs. Please clarify the final annotated gene content and ensure that the Results, figures, and Conclusion are fully consistent."

**Response:** We have corrected the Results to show 22 tRNAs.

**Reviewer #4:** "The maximum-likelihood analyses were run with 100 bootstrap replicates only, which is relatively modest for a study making several topological comparisons across datasets."

**Response:** Phylogenetic analysis methodology is now overhauled. Node support was assessed using 1000 ultrafast bootstrap replicates (UFBoot2) and 1000 SH-aLRT replicates.

**Reviewer #4:** "The phylogenetic Methods section states that 'the protein model' with the lowest AIC/BIC was chosen, but the trees were then inferred using nucleotide substitution models such as GTR+G+I and TN93+G+I. Please clarify whether the analyses were performed on nucleotide alignments, amino-acid alignments, or both, and revise the model-selection description accordingly."

**Response:** Phylogenetic analysis methodology was overhauled. The original trees were reconstructed using IQ-TREE2 with a fully partitioned analysis framework. For the protein-coding gene (PCG) datasets, each gene was partitioned by codon position into first and second codon positions (pos12) and third codon position (pos3) as separate subsets. Ribosomal RNA genes (rrnS and rrnL) were each treated as a single partition. Model selection was performed independently for each partition using ModelFinder implemented in IQ-TREE2, with the best-fit substitution model selected under the Bayesian Information Criterion (BIC).

**Reviewer #4:** "Table 1 appears to contain a classification problem because Circaetus pectoralis is listed under 'Cathartidae,' which is inconsistent with the broader structure of the table and with the manuscript's own discussion of Circaetinae."

**Response:** We have corrected this to say Circaetinae.

**Reviewer #4:** "Please replace 'singular mitochondrial genes' with 'single mitochondrial genes,' which is the more natural term in this context."

**Response:** Corrected as suggested throughout the manuscript.
