# Supplementary material for: The mitochondrial genomes of the endemic and endangered South Philippine Hawk-eagle Nisaetus pinskeri and other Nisaetus Hawk-eagles (Aves, Accipitriformes)
Source: PLoS One. 2026 Oct 26;21(10):e0360538. doi: 10.1371/journal.pone.0360538 (PMC13704876; doi:10.1371/journal.pone.0360538)
Supplement: Peer review history

# Peer review: Decision Letter 0

**DOI:** 10.1371/journal.pone.0360538.r001

**Authors:** James Lee Crainey

11 May 2026

Dear Dr. Perdon,

Thank you for submitting your manuscript to PLOS ONE. After careful consideration, we feel that it has merit but does not fully meet PLOS ONE’s publication criteria as it currently stands. Therefore, we invite you to submit a revised version of the manuscript that addresses the points raised during the review process.

Please accept my apologies for the delay in getting this first decision to you. Your reviewers provided conflicting recommendations, so I wanted to have a higher number of reports (than usual) before taking my decision. As you will see from these reports, the reviewers do not have many concerns about the novelty and value of your data and only very limited concerns about the way you have analyzed your data. Most concerns are related to a perceived over interpretation of your data. In my view this can be dealt with by reducing the length of your discussion and restricting your speculation about the significance of your findings (focusing specifically on what you have found without much extrapolation on what that might mean). Two of your reviewers specifically raised concerns about the claims that your mitogenomes are complete. For your work to be published in PLOS ONE you must address this concern by either: (1) changing how your refer to your mitogenomes (i.e as draft genomes, or near-complete genomes et cetera) or (2) by providing additional supporting data justifying your complete genome claim or (3) by rebutting the reviewers´ criticisms with clear reasoned justifications for your use of the term “complete” mitogenomes.

Please submit your revised manuscript by Jun 25 2026 11:59PM. If you will need more time than this to complete your revisions, please reply to this message or contact the journal office at plosone@plos.org. When you're ready to submit your revision, log on to https://www.editorialmanager.com/pone/ and select the 'Submissions Needing Revision' folder to locate your manuscript file.

- A letter that responds to each point raised by the academic editor and reviewer(s). You should upload this letter as a separate file labeled 'Response to Reviewers'.
- A marked-up copy of your manuscript that highlights changes made to the original version. You should upload this as a separate file labeled 'Revised Manuscript with Track Changes'.
- An unmarked version of your revised paper without tracked changes. You should upload this as a separate file labeled 'Manuscript'.

If you would like to make changes to your financial disclosure, please include your updated statement in your cover letter. Guidelines for resubmitting your figure files are available below the reviewer comments at the end of this letter.

If applicable, we recommend that you deposit your laboratory protocols in protocols.io to enhance the reproducibility of your results. Protocols.io assigns your protocol its own identifier (DOI) so that it can be cited independently in the future. For instructions see: https://journals.plos.org/plosone/s/submission-guidelines#loc-laboratory-protocols. Additionally, PLOS ONE offers an option for publishing peer-reviewed Lab Protocol articles, which describe protocols hosted on protocols.io. Read more information on sharing protocols at https://plos.org/protocols?utm_medium=editorial-email&utm_source=authorletters&utm_campaign=protocols.

As the corresponding author, your ORCID iD is verified in the submission system and will appear in the published article. PLOS supports the use of ORCID, and we encourage all coauthors to register for an ORCID iD and use it as well. Please encourage your coauthors to verify their ORCID iD within the submission system before final acceptance, as unverified ORCID iDs will not appear in the published article. *Only*  the individual author can complete the verification step; PLOS staff *cannot*  verify ORCID iDs on behalf of authors.

We look forward to receiving your revised manuscript.

Kind regards,

James Lee Crainey, Ph.D.

Academic Editor

PLOS One

Journal Requirements:

When submitting your revision, we need you to address these additional requirements.

1. Please ensure that your manuscript meets PLOS ONE's style requirements, including those for file naming. The PLOS ONE style templates can be found at

https://journals.plos.org/plosone/s/file?id=wjVg/PLOSOne_formatting_sample_main_body.pdf and

https://journals.plos.org/plosone/s/file?id=ba62/PLOSOne_formatting_sample_title_authors_affiliations.pdf

2. To comply with PLOS One submissions requirements, in your Methods section, please provide additional information regarding the experiments involving animals and ensure you have included details on (1) methods of sacrifice, (2) methods of anesthesia and/or analgesia, and (3) efforts to alleviate suffering.

3. Thank you for stating the following in the Acknowledgments Section of your manuscript:

"We also thank the Philippine Genome Center, University of the Philippines System, for their partial financial support, and MGI for supplying the DNBSEQ sequencing kits."

We note that you have provided additional information within the Acknowledgements Section that is not currently declared in your Funding Statement. Please note that funding information should not appear in the Acknowledgments section or other areas of your manuscript. We will only publish funding information present in the Funding Statement section of the online submission form.

Please remove any funding-related text from the manuscript and let us know how you would like to update your Funding Statement. Currently, your Funding Statement reads as follows:

"The author(s) received no specific funding for this work.

"Please include your amended statements within your cover letter; we will change the online submission form on your behalf.

4. Thank you for stating the following financial disclosure:

"The author(s) received no specific funding for this work."

At this time, please address the following queries:

a)        Please clarify the sources of funding (financial or material support) for your study. List the grants or organizations that supported your study, including funding received from your institution.

b)        State what role the funders took in the study. If the funders had no role in your study, please state: “The funders had no role in study design, data collection and analysis, decision to publish, or preparation of the manuscript.”

c)        If any authors received a salary from any of your funders, please state which authors and which funders.

d)        If you did not receive any funding for this study, please state: “The authors received no specific funding for this work.”

Please include your amended statements within your cover letter; we will change the online submission form on your behalf.

5. Please ensure that you refer to Figures 4, 5 and 7 in your text as, if accepted, production will need this reference to link the reader to the figure.

6. Please note that your Data Availability Statement is currently missing a direct link to access each database. If your manuscript is accepted for publication, you will be asked to provide these details on a very short timeline. We therefore suggest that you provide this information now, though we will not hold up the peer review process if you are unable.

7. Please include captions for your Supporting Information files at the end of your manuscript, and update any in-text citations to match accordingly. Please see our Supporting Information guidelines for more information: http://journals.plos.org/plosone/s/supporting-information.

8. If the reviewer comments include a recommendation to cite specific previously published works, please review and evaluate these publications to determine whether they are relevant and should be cited. There is no requirement to cite these works unless the editor has indicated otherwise.

9. We note that Figure 1 in your submission contain [map/satellite] images which may be copyrighted. All PLOS content is published under the Creative Commons Attribution License (CC BY 4.0), which means that the manuscript, images, and Supporting Information files will be freely available online, and any third party is permitted to access, download, copy, distribute, and use these materials in any way, even commercially, with proper attribution. For these reasons, we cannot publish previously copyrighted maps or satellite images created using proprietary data, such as Google software (Google Maps, Street View, and Earth). For more information, see our copyright guidelines: http://journals.plos.org/plosone/s/licenses-and-copyright.

We require you to either (1) present written permission from the copyright holder to publish these figures specifically under the CC BY 4.0 license, or (2) remove the figures from your submission:

a. You may seek permission from the original copyright holder of Figure 1 to publish the content specifically under the CC BY 4.0 license.

We recommend that you contact the original copyright holder with the Content Permission Form (http://journals.plos.org/plosone/s/file?id=7c09/content-permission-form.pdf) and the following text:

“I request permission for the open-access journal PLOS ONE to publish XXX under the Creative Commons Attribution License (CCAL) CC BY 4.0 (http://creativecommons.org/licenses/by/4.0/). Please be aware that this license allows unrestricted use and distribution, even commercially, by third parties. Please reply and provide explicit written permission to publish XXX under a CC BY license and complete the attached form.”

Please upload the completed Content Permission Form or other proof of granted permissions as an "Other" file with your submission.

In the figure caption of the copyrighted figure, please include the following text: “Reprinted from [ref] under a CC BY license, with permission from [name of publisher], original copyright [original copyright year].”

b. If you are unable to obtain permission from the original copyright holder to publish these figures under the CC BY 4.0 license or if the copyright holder’s requirements are incompatible with the CC BY 4.0 license, please either i) remove the figure or ii) supply a replacement figure that complies with the CC BY 4.0 license. Please check copyright information on all replacement figures and update the figure caption with source information. If applicable, please specify in the figure caption text when a figure is similar but not identical to the original image and is therefore for illustrative purposes only.

The following resources for replacing copyrighted map figures may be helpful:

USGS National Map Viewer (public domain): http://viewer.nationalmap.gov/viewer/

The Gateway to Astronaut Photography of Earth (public domain): http://eol.jsc.nasa.gov/sseop/clickmap/

Maps at the CIA (public domain): https://www.cia.gov/library/publications/the-world-factbook/index.html and https://www.cia.gov/library/publications/cia-maps-publications/index.html

NASA Earth Observatory (public domain): http://earthobservatory.nasa.gov/

Landsat: http://landsat.visibleearth.nasa.gov/

USGS EROS (Earth Resources Observatory and Science (EROS) Center) (public domain): http://eros.usgs.gov/#

Natural Earth (public domain): http://www.naturalearthdata.com/

[Note: HTML markup is below. Please do not edit.]

Reviewers' comments:

Reviewer's Responses to Questions

**Comments to the Author**

1. Is the manuscript technically sound, and do the data support the conclusions?

Reviewer #1: Yes

Reviewer #2: Partly

Reviewer #3: Partly

Reviewer #4: Partly

**********

2. Has the statistical analysis been performed appropriately and rigorously?

Reviewer #1: Yes

Reviewer #2: N/A

Reviewer #3: I Don't Know

Reviewer #4: Yes

**********

3. Have the authors made all data underlying the findings in their manuscript fully available?

The PLOS Data policy

Reviewer #1: Yes

Reviewer #2: No

Reviewer #3: No

Reviewer #4: No

**********

4. Is the manuscript presented in an intelligible fashion and written in standard English?

Reviewer #1: Yes

Reviewer #2: Yes

Reviewer #3: Yes

Reviewer #4: Yes

**********

Reviewer #1: This manuscript reports a new mitochondrial genome from an interesting and endangered bird species. The sequencing, assembly, and downstream analyses appear technically sound, detailed, and follow established standards. I have no major methodological concerns, except that some analyses are interesting, but might be an overkill (FIG 3 and 4).

However, the study is largely descriptive, consisting of genome assembly, annotation, and routine phylogenetic analysis, without providing additional biological insight or broader implications. As such, the contribution is limited in scope and does not meet the level of general interest typically expected for PLOS ONE.

In my view, the manuscript would be better suited for a specialized resource or data-focused journal, such as Mitochondrial DNA Part B: Resources or Data in Brief, where such contributions are within scope.

Alt:

PeerJ

Data in Brief

ZooKeys

May be a better match and easier to publish in.

Reviewer #2: The manuscript reports the mitochondrial genomes of the southern Philippine endemic and endangered Nisaetus pinskeri and Nisaetus cirrhatus, and compares them with mitochondrial genomes of other Accipitriformes birds. The study has certain conservation biological significance, particularly because N. pinskeri is a regionally endemic and endangered raptor. Its mitochondrial genome data may provide a useful reference for future studies on genetic diversity, phylogeny, and conservation genetics. Therefore, this study has some resource value and publication potential. However, in its current form, the manuscript still has several important issues, especially regarding the reliability of mitochondrial genome assembly, the methodological rigor of the phylogenetic analyses, whether the results sufficiently support the conclusions, and whether the conservation implications are overstated. The most serious issue is that the authors clearly state that the mitochondrial genome of N. pinskeri was obtained by manually concatenating two contigs, while also noting that the control region and pseudo-control region have low coverage and contain repeat-rich regions. In the absence of evidence from long-range PCR, Sanger sequencing, long-read sequencing, junction-spanning reads, or assembly graphs, the current evidence is insufficient to support the claim that it represents a “complete mitochondrial genome”. Therefore, the authors should either provide additional validation evidence or describe this mitogenome more cautiously throughout the manuscript as “near-complete”, “tentative”, or “putatively assembled”. Overall, the manuscript could serve as a mitochondrial genome resource study, but the current version does not sufficiently support its stronger phylogenetic and conservation biological conclusions.

1 The authors do not provide a sufficiently comprehensive review of existing molecular phylogenetic studies on the genus Nisaetus. In particular, the taxonomic history of N. pinskeri, which was previously treated as a subspecies of N. philippensis, and the support from existing cytb and control-region sequences for distinguishing these taxa should be more clearly described in the Introduction. Second, although the authors emphasize that complete mitochondrial genomes provide higher resolution than single-gene markers, they do not adequately discuss the inherent limitations of mitochondrial data, such as maternal inheritance, representation of a single genetic background, lack of recombination, and possible discordance with nuclear genomic phylogenies. Third, the conservation biological significance is stated rather broadly. One or a few mitochondrial genomes can provide useful reference sequences, but they cannot directly reveal population genetic diversity, levels of inbreeding, effective population size, or conservation management units.

2 The authors should at least provide the following information: the number of raw reads, the number of filtered reads, the Q30 percentage, the average mitochondrial coverage, the minimum coverage, contig lengths, the repeat-sequence features at the contig ends, the specific basis for manual concatenation, and whether junction-spanning reads support the inferred connection. In addition, the authors are encouraged to use other mitochondrial genome assembly programs, such as MitoZ, GetOrganelle, or MitoFinder, for cross-validation. If no experimental validation or additional assembly evidence can be provided, the term “complete mitogenome” should be avoided.

Regarding the selection pressure analysis, the authors used SLAC to calculate Ka/Ks, but the methodological description is insufficient. They should clarify whether the input files were codon alignments, whether each gene was analyzed separately, whether a phylogenetic tree was used, and whether stop codons and anomalous insertion sites were removed.

3 The Discussion is somewhat unfocused. Some sections, such as the reclassification of the genus Accipiter, the taxonomic controversy between Cathartiformes and Accipitriformes, and the possible mislabeling of the Nisaetus floris sample, are interesting but not closely connected to the core contribution of this manuscript. These side discussions should be condensed, and the Discussion should focus more clearly on the two newly assembled mitochondrial genomes, the uncertainty in control-region structure, comparisons within Nisaetus, and the value of these data for future research.

4 This manuscript may be more suitable for a journal that specifically publishes mitochondrial genome resource or genome data papers.

Reviewer #3: Comments to the Authors

This manuscript reports mitogenomic data for Nisaetus pinskeri and Nisaetus cirrhatus and provides comparative analyses with other Accipitriformes/Cathartiformes mitogenomes. Nisaetus pinskeri is an endemic and endangered raptor from the Philippines, and mitogenomic resources for this species may be useful for future conservation genetic studies. However, the current manuscript is closer to a mitogenomic resource report with routine comparative analyses, and several key claims are not sufficiently supported.

The most serious issue is that the N. pinskeri mitogenome was assembled as two contigs and then manually concatenated, but the manuscript does not provide sufficient technical details to explain how this manual joining was performed. For example, it is unclear whether the connection between the two contigs was supported by overlapping sequences, read mapping across the junctions, paired-end read information, PCR/Sanger validation, or other independent evidence. This lack of detail raises concerns about the reproducibility and reliability of the reported circular mitogenome. Therefore, the claim of a “complete mitogenome” requires additional validation or more cautious wording.

Second, the phylogenetic analyses also require substantial revision. The manuscript appears to use only 100 bootstrap replicates, which is insufficient for supporting the phylogenetic conclusions drawn here. In addition, the basis for choosing the reported models, including GTR+G+I and TN93+G+I, is not clearly justified. These models should be selected using an appropriate model-selection procedure, such as ModelFinder or a comparable tool, rather than being applied without explanation. The authors should also clarify whether the mitochondrial protein-coding genes were partitioned by gene and/or codon position, whether RNA genes and protein-coding genes were modeled separately when combined, and whether amino-acid-based analyses were tested. Without a more rigorous phylogenetic framework, including appropriate model selection, partitioned analyses, and at least 1000 bootstrap or ultrafast bootstrap replicates, the resulting topology and the evolutionary interpretations based on it are not sufficiently reliable.

Major comments

1. The claim of a “complete mitogenome” for N. pinskeri is not sufficiently supported

The authors state that the mitogenome assembly of N. pinskeri using NOVOPlasty resulted in two contigs, which were subsequently manually concatenated. I do not consider the failure to recover a single circular molecule using NOVOPlasty alone to be unusual, particularly for repeat-rich mitochondrial regions. However, because the control region and pseudo-control region appear to have low coverage and contain repetitive sequences, the final structure and length of the N. pinskeri mitogenome remain uncertain.

The authors should provide additional evidence supporting the manually joined assembly, especially around the contig junctions, control region, pseudo-control region, and repeat-rich regions. Ideally, the assembly should be validated using long-range PCR/Sanger sequencing or long-read sequencing. If such validation cannot be provided, the authors should avoid repeatedly using “complete mitogenome” or “complete mitochondrial genome” and instead use more cautious wording, such as “near-complete mitochondrial genome” or “draft mitochondrial genome”. All conclusions based on genome length, repeat number, and control-region structure should also be weakened accordingly.

2. Additional assembly validation and sequencing-depth plots are needed

Because the control region and pseudo-control region have low coverage and repetitive sequences, the authors should test additional mitogenome assembly tools, such as MitoZ, MitoFinder, GetOrganelle, or other suitable assemblers, to determine whether the mitogenome can be independently recovered as a circular molecule. This is particularly important because manual concatenation of contigs is not easily reproducible and may introduce uncertainty in repeat-rich regions.

The authors should also provide sequencing-depth plots across the entire mitogenome, with particular attention to the manually joined contig boundaries, control region, pseudo-control region, and repeat-rich regions. The statement that read depth was estimated using MitoZ is not sufficiently transparent. Although MitoZ may provide coverage-related information, the authors should map the raw reads back to the assembled mitogenome using a standard approach, such as BWA, Bowtie2, or minimap2, and calculate coverage using SAMtools depth, SAMtools coverage, or bedtools genomecov.

3. The annotation procedure needs clarification

The authors state that MITOS2 was used for mitogenome annotation and that tRNAs were verified using tRNAscan-SE v2.0. However, it is unclear whether the tRNA annotations inferred by tRNAscan-SE were fully consistent with those generated by MITOS2. Inconsistencies between these two tools are common in mitochondrial genome annotation, and the authors should briefly explain how the final tRNA annotations were determined.

4. The alignment strategy should be revised

Different gene types should not necessarily be aligned using the same approach. For mitochondrial protein-coding genes, the authors should use an amino-acid-guided or codon-aware alignment strategy, rather than simply aligning nucleotide sequences directly. For example, PCGs could be translated into amino acids, aligned at the protein level, and then back-translated to codon alignments for downstream nucleotide-based phylogenetic or selection analyses.

Ribosomal RNA genes and tRNA genes should be aligned separately using methods appropriate for RNA sequences. The authors should clearly describe how each class of genes was aligned, trimmed, and concatenated.

5. The phylogenetic analyses require substantial revision

The use of only 100 bootstrap replicates is clearly insufficient for the phylogenetic conclusions drawn in this manuscript. The authors should reconstruct the phylogenetic trees using a more rigorous framework, such as IQ-TREE or MrBayes, with appropriate model selection, partitioned analyses, and at least 1000 ultrafast bootstrap or standard bootstrap replicates.

The authors should also clarify whether the 13 mitochondrial PCGs were partitioned by gene and/or codon position, whether third codon positions were separately modeled or excluded, and whether amino-acid datasets were tested. These choices can strongly influence mitogenomic phylogenetic inference.

Furthermore, the manuscript mentions a “protein model”, but then reports the use of GTR+G+I and TN93+G+I models. This is confusing and possibly incorrect. The authors should clarify the basis for choosing these models and whether the same model was applied to all three datasets. When RNA genes and PCGs are combined in the same analysis, the appropriateness of the model and partitioning scheme should also be justified.

6. The mitochondrial phylogeny should not be overinterpreted

A mitochondrial genome represents a single maternally inherited linkage unit and cannot by itself represent the species tree. The authors should avoid overinterpreting the mitochondrial topology, especially for higher-level relationships within Accipitriformes.

The phrase “basal taxa of Accipitriformes” should also be avoided. Extant taxa are not “basal” in a strict phylogenetic sense; particular lineages may be inferred as early-diverging relative to other sampled lineages in a given topology. A more appropriate wording would be “early-diverging lineages within Accipitriformes, including Pandionidae and Sagittariidae.”

7. The extra cytosine insertion in nad3 requires further explanation

The reported extra cytosine at position 174 of the nad3 gene is interesting. However, the authors should clearly explain how this insertion was handled during translation, sequence alignment, and dN/dS analysis. Since this insertion may cause an apparent frameshift but is known in birds and may involve translational frameshifting or RNA editing, it is important to clarify whether the insertion was retained, removed, or manually corrected in each downstream analysis.

8. The selection-pressure analysis is descriptive and overinterpreted

The authors used SLAC/Datamonkey to calculate Ka/Ks or dN/dS values and found that all mitochondrial PCGs are under purifying selection. This result is expected for avian mitochondrial protein-coding genes and does not provide evidence for species-specific adaptation in N. pinskeri or N. cirrhatus.

A single overall dN/dS value per gene across Accipitridae or Accipitriformes only indicates general conservation; it does not support claims regarding environmental adaptation or conservation-relevant functional evolution. Similarly, the finding that atp8 has the highest dN/dS and cox1 the lowest is a common pattern and should not be presented as a novel biological insight unless supported by additional comparative analyses.

If the authors wish to discuss adaptive evolution, more appropriate branch-based or branch-site analyses would be required. Otherwise, this section should be presented as a descriptive comparison of mitochondrial gene conservation.

9. Conservation implications should be weakened

The manuscript repeatedly suggests that these mitogenomes can provide critical insights for genetic diversity monitoring and conservation management. However, the current study includes only one or a few mitogenomes and does not include population-level sampling, haplotype diversity, effective population size, inbreeding, genetic bottlenecks, or population connectivity analyses.

Therefore, the manuscript cannot directly support conclusions about conservation strategies. The authors should state more cautiously that these mitogenomes provide reference sequences for future population genetic, phylogeographic, and conservation genomic studies.

10. Fig. 3 is difficult to interpret and should be improved or replaced

Fig. 3 is difficult to interpret in its current form. The individual rings are not directly labeled, and readers must infer the identity of each ring from the figure caption. More importantly, the most variable regions highlighted in this figure are the control region and pseudo-control region, which are also the regions affected by low coverage and repeat-resolution uncertainty.

Therefore, the current visualization does not adequately support the subsequent discussion of length variation, repeat regions, and control-region structure. The authors should either redesign Fig. 3 with clearer ring labels and a more informative legend, or consider replacing it with a more interpretable visualization method.

Minor comments

1. The title is quite long and could be simplified.

2. The abstract should avoid overly strong phrases such as “critical insights” unless the conclusions are directly supported by the analyses.

3. Please ensure consistency in the reported length of the N. cirrhatus mitogenome. The abstract reports 17,873 bp, whereas the results section reports 17,874 bp.

4. The terms “Changeable Hawk-eagle” and “Pinsker’s Hawk-eagle/South Philippine Hawk-eagle” should be used consistently throughout the manuscript.

5. The phrase “family Accipitriformes” should be corrected, as Accipitriformes is an order, not a family.

6. “itsadaptability” appears to lack a space.

7. The attempted assembly of the Nisaetus floris dataset and its subsequent identification as likely Aquila fasciata are potentially important, but this part is currently unclear. The authors should clarify whether this re-identification is part of the present study and, if so, what evidence supports it. Was it based on the complete mitogenome, particular mitochondrial genes, genome-wide data, specimen metadata, or museum records? The authors should also state whether the N. floris dataset was excluded from all downstream analyses and whether this potential misidentification has been reported to the relevant database or museum collection.

8. Figure legends should be expanded to make the figures easier to understand without excessive reliance on the main text.

9. The manuscript should clearly distinguish between newly generated data, publicly available WGS data reassembled in this study, and previously published RefSeq mitogenomes.

Overall recommendation

The manuscript contains potentially useful mitogenomic data for an endangered raptor, but the current version has major problems in assembly validation, reproducibility, phylogenetic methodology, and interpretation. Substantial revision, additional validation, and reanalysis are required before the manuscript can be considered for publication.

Reviewer #4: This study addresses a relevant topic and provides useful mitochondrial genomic information for Nisaetus hawk-eagles, especially for the endangered Nisaetus pinskeri. However, I think the manuscript requires revision before it can be considered technically sound in full

**********

what does this mean?). If published, this will include your full peer review and any attached files.

If you choose “no”, your identity will remain anonymous but your review may still be made public.

**Do you want your identity to be public for this peer review?**  For information about this choice, including consent withdrawal, please see our Privacy Policy

Reviewer #1: **Yes:**  Axel Janke

Reviewer #2: No

Reviewer #3: No

Reviewer #4: **Yes:**  Dwi Sendi Priyono

**********

[NOTE: If reviewer comments were submitted as an attachment file, they will be attached to this email and accessible via the submission site. Please log into your account, locate the manuscript record, and check for the action link "View Attachments". If this link does not appear, there are no attachment files.]

To ensure your figures meet our technical requirements, please review our figure guidelines: https://journals.plos.org/plosone/s/figures

You may also use PLOS’s free figure tool, NAAS, to help you prepare publication quality figures: https://journals.plos.org/plosone/s/figures#loc-tools-for-figure-preparation.

NAAS will assess whether your figures meet our technical requirements by comparing each figure against our figure specifications.

Attachment Submitted filename: reviewer PLOSONE nisaetus.docx

# Author comment: Author response to Decision Letter 1

**DOI:** 10.1371/journal.pone.0360538.r002

3 Sep 2026

Response to the Academic Editor

Editor Comment: "As you will see from these reports, the reviewers do not have many concerns about the novelty and value of your data and only very limited concerns about the way you have analyzed your data. Most concerns are related to a perceived over interpretation of your data. In my view this can be dealt with by reducing the length of your discussion and restricting your speculation about the significance of your findings (focusing specifically on what you have found without much extrapolation on what that might mean)."

Response: We thank the Editor for this guidance. We have substantially revised the Results, Discussion, and Conclusion to focus on describing our findings directly, removing speculative extrapolation regarding adaptive significance and conservation implications not directly supported by our data.

Editor Comment: "Two of your reviewers specifically raised concerns about the claims that your mitogenomes are complete. For your work to be published in PLOS ONE you must address this concern by either: (1) changing how your refer to your mitogenomes (i.e as draft genomes, or near-complete genomes et cetera) or (2) by providing additional supporting data justifying your complete genome claim or (3) by rebutting the reviewers´ criticisms with clear reasoned justifications for your use of the term "complete" mitogenomes."

Response: We have switched to "near-complete" or "draft" mitogenome when referring to both the Nisaetus pinskeri and Nisaetus cirrhatus throughout the whole paper.

Response to Reviewer #1

Reviewer #1: "This manuscript reports a new mitochondrial genome from an interesting and endangered bird species. The sequencing, assembly, and downstream analyses appear technically sound, detailed, and follow established standards. I have no major methodological concerns, except that some analyses are interesting, but might be an overkill (FIG 3 and 4)."

Response: We thank the reviewer for this positive assessment of our methodology. We have revised Figure 3 to improve its interpretability. We have retained Figure 4 (RSCU analysis) as codon usage bias is a standard component of mitogenome resource papers and provides useful reference data for future comparative studies, but have shortened the accompanying text to a more descriptive treatment.

Response to Reviewer #2

Reviewer #2: "The manuscript reports the mitochondrial genomes of the southern Philippine endemic and endangered Nisaetus pinskeri and Nisaetus cirrhatus, and compares them with mitochondrial genomes of other Accipitriformes birds. The study has certain conservation biological significance. Therefore, this study has some resource value and publication potential. However, in its current form, the manuscript still has several important issues, especially regarding the reliability of mitochondrial genome assembly, the methodological rigor of the phylogenetic analyses, whether the results sufficiently support the conclusions, and whether the conservation implications are overstated."

Response: We thank the reviewer for this assessment and address each specific issue raised below.

Reviewer #2: "The most serious issue is that the authors clearly state that the mitochondrial genome of N. pinskeri was obtained by manually concatenating two contigs, while also noting that the control region and pseudo-control region have low coverage and contain repeat-rich regions. In the absence of evidence from long-range PCR, Sanger sequencing, long-read sequencing, junction-spanning reads, or assembly graphs, the current evidence is insufficient to support the claim that it represents a "complete mitochondrial genome". Therefore, the authors should either provide additional validation evidence or describe this mitogenome more cautiously throughout the manuscript as "near-complete", "tentative", or "putatively assembled"."

Response: We have switched to "near-complete" or "draft" mitogenome when referring to both the Nisaetus pinskeri and Nisaetus cirrhatus throughout the whole paper.

Reviewer #2: "The authors do not provide a sufficiently comprehensive review of existing molecular phylogenetic studies on the genus Nisaetus. In particular, the taxonomic history of N. pinskeri, which was previously treated as a subspecies of N. philippensis, and the support from existing cytb and control-region sequences for distinguishing these taxa should be more clearly described in the Introduction."

Response: We have added this part: “Likewise, N. pinskeri was formerly considered a subspecies of the Philippine hawk-eagle (N. philippensis) but was elevated to species status following a mitochondrial study by Gamauf et al., also using cytb and control region sequences, that demonstrated clear genetic differentiation, likely resulting from an early Pleistocene geographic isolation between the Greater Luzon and Greater Mindanao faunal regions” in the Introduction.

Reviewer #2: "Second, although the authors emphasize that complete mitochondrial genomes provide higher resolution than single-gene markers, they do not adequately discuss the inherent limitations of mitochondrial data, such as maternal inheritance, representation of a single genetic background, lack of recombination, and possible discordance with nuclear genomic phylogenies."

Response: We have added this part: “Nevertheless, the maternal inheritance and lack of recombination of mtDNA mean that mitogenomes represent a single evolutionary history, which may not always reflect species relationships.” in the Introduction.

Reviewer #2: "Third, the conservation biological significance is stated rather broadly. One or a few mitochondrial genomes can provide useful reference sequences, but they cannot directly reveal population genetic diversity, levels of inbreeding, effective population size, or conservation management units."

Response: We have revised the Discussion and Conclusion to state that these mitogenomes serve as reference sequences for future population genetic, phylogeographic, and conservation genomic studies, rather than as direct sources of conservation-management conclusions.

Reviewer: "The authors should at least provide the following information: the number of raw reads, the number of filtered reads, the Q30 percentage, the average mitochondrial coverage, the minimum coverage, contig lengths, the repeat-sequence features at the contig ends, the specific basis for manual concatenation, and whether junction-spanning reads support the inferred connection."

Response: We added “Quality control using Fastp yielded a total of 65.09 G raw reads, of which 64.77 G remained after filtering, with a Q30 percentage of 92.96%, providing the basis for subsequent mitochondrial assembly and coverage analyses.” at the start of the results. We also added S2 Figure to show the repeat-sequence features at the contig ends and the sequence depth plot.

Reviewer #2: "In addition, the authors are encouraged to use other mitochondrial genome assembly programs, such as MitoZ, GetOrganelle, or MitoFinder, for cross-validation. If no experimental validation or additional assembly evidence can be provided, the term "complete mitogenome" should be avoided."

Response: We have performed the additional analyses using the three suggested assemblers and have provided the output in S1 Figure. We have deemed that the NOVOPlasty output still has the most merit. We have avoided the term “complete mitogenome” throughout the revised manuscript.

Reviewer #2: "Regarding the selection pressure analysis, the authors used SLAC to calculate Ka/Ks, but the methodological description is insufficient. They should clarify whether the input files were codon alignments, whether each gene was analyzed separately, whether a phylogenetic tree was used, and whether stop codons and anomalous insertion sites were removed."

Response: We have added “which requires codon-based multiple sequence alignments and a corresponding phylogenetic tree as input (see Mitogenomic phylogenetic analysis)” to the methodology to clarify the input files. Stop codons and insertions were removed.

Reviewer #2: "The Discussion is somewhat unfocused. Some sections, such as the reclassification of the genus Accipiter, the taxonomic controversy between Cathartiformes and Accipitriformes, and the possible mislabeling of the Nisaetus floris sample, are interesting but not closely connected to the core contribution of this manuscript. These side discussions should be condensed, and the Discussion should focus more clearly on the two newly assembled mitochondrial genomes, the uncertainty in control-region structure, comparisons within Nisaetus, and the value of these data for future research."

Response: We have condensed the sections on Accipiter reclassification and the Cathartiformes/Accipitriformes taxonomic controversy to brief, single-sentence mentions with citation, and refocused the Discussion on the two newly assembled genomes, control-region structural uncertainty, within-Nisaetus comparisons, and future research value, per the reviewer's suggestion.

Response to Reviewer #3

Reviewer #3: "This manuscript reports mitogenomic data for Nisaetus pinskeri and Nisaetus cirrhatus and provides comparative analyses with other Accipitriformes/Cathartiformes mitogenomes... However, the current manuscript is closer to a mitogenomic resource report with routine comparative analyses, and several key claims are not sufficiently supported."

Response: We thank the reviewer for this assessment and address the specific concerns below.

Reviewer #3: "The most serious issue is that the N. pinskeri mitogenome was assembled as two contigs and then manually concatenated, but the manuscript does not provide sufficient technical details to explain how this manual joining was performed... Therefore, the claim of a "complete mitogenome" requires additional validation or more cautious wording."

Response: We have switched to "near-complete" or "draft" mitogenome when referring to both the Nisaetus pinskeri and Nisaetus cirrhatus throughout the whole paper.

Reviewer #3: "Second, the phylogenetic analyses also require substantial revision. The manuscript appears to use only 100 bootstrap replicates, which is insufficient... the basis for choosing the reported models, including GTR+G+I and TN93+G+I, is not clearly justified. These models should be selected using an appropriate model-selection procedure, such as ModelFinder or a comparable tool... Without a more rigorous phylogenetic framework, including appropriate model selection, partitioned analyses, and at least 1000 bootstrap or ultrafast bootstrap replicates, the resulting topology and the evolutionary interpretations based on it are not sufficiently reliable."

Response: The original trees were reconstructed using IQ-TREE2 with a fully partitioned analysis framework. For the protein-coding gene (PCG) datasets, each gene was partitioned by codon position into first and second codon positions (pos12) and third codon position (pos3) as separate subsets. Ribosomal RNA genes (rrnS and rrnL) were each treated as a single partition. Model selection was performed independently for each partition using ModelFinder implemented in IQ-TREE2, with the best-fit substitution model selected under the Bayesian Information Criterion (BIC). Partition schemes were further optimized by merging subsets with statistically indistinguishable models using the greedy merging algorithm in ModelFinder, reducing the risk of overparameterization. The GTR+G+I and TN93+G+I models used in the original manuscript were replaced by partition-specific models selected through this procedure, and are now reported in full in the manuscript. Node support was assessed using 1000 ultrafast bootstrap replicates (UFBoot2) and 1000 SH-aLRT replicates.

To address the reviewer's concern regarding amino acid datasets, each PCG alignment was also translated using the vertebrate mitochondrial genetic code (NCBI translation table 2) and analyzed as a separate amino acid dataset, with model selection restricted to mitochondrial substitution matrices (mtREV, mtZOA, mtMAM, mtART) alongside general protein models (WAG and LG). The amino acid-based topology was consistent with the nucleotide-based partitioned trees, supporting the robustness of the inferred relationships.

The revised methods, updated model assignments per partition, and revised phylogenetic trees are reflected in the updated manuscript.

Reviewer #3: "The claim of a "complete mitogenome" for N. pinskeri is not sufficiently supported... The authors should provide additional evidence supporting the manually joined assembly, especially around the contig junctions, control region, pseudo-control region, and repeat-rich regions. Ideally, the assembly should be validated using long-range PCR/Sanger sequencing or long-read sequencing. If such validation cannot be provided, the authors should avoid repeatedly using "complete mitogenome" or "complete mitochondrial genome" and instead use more cautious wording... All conclusions based on genome length, repeat number, and control-region structure should also be weakened accordingly."

Response: We have switched to "near-complete" or "draft" mitogenome when referring to both the Nisaetus pinskeri and Nisaetus cirrhatus throughout the whole paper.

Reviewer #3: "Because the control region and pseudo-control region have low coverage and repetitive sequences, the authors should test additional mitogenome assembly tools, such as MitoZ, MitoFinder, GetOrganelle... The authors should also provide sequencing-depth plots across the entire mitogenome... The statement that read depth was estimated using MitoZ is not sufficiently transparent. Although MitoZ may provide coverage-related information, the authors should map the raw reads back to the assembled mitogenome using a standard approach, such as BWA, Bowtie2, or minimap2, and calculate coverage using SAMtools depth, SAMtools coverage, or bedtools genomecov."

Response: We have performed the additional analyses using the three suggested assemblers and have provided the output in S1 Figure. We have deemed that the NOVOPlasty output still has the most merit. We also added S2 Figure to show the repeat-sequence features at the contig ends and the sequence depth plots.

Reviewer #3: "It is unclear whether the tRNA annotations inferred by tRNAscan-SE were fully consistent with those generated by MITOS2. Inconsistencies between these two tools are common in mitochondrial genome annotation, and the authors should briefly explain how the final tRNA annotations were determined."

Response: All tRNA genes were independently verified in an independent annotation with tRNAscan-SE.

Reviewer #3: "For mitochondrial protein-coding genes, the authors should use an amino-acid-guided or codon-aware alignment strategy... Ribosomal RNA genes and tRNA genes should be aligned separately using methods appropriate for RNA sequences. The authors should clearly describe how each class of genes was aligned, trimmed, and concatenated."

Response: We have revised the alignment strategy accordingly. Protein-coding genes were aligned using MACSE v2, which performs codon-aware alignment directly on nucleotide sequences while accounting for frameshifts, using the vertebrate mitochondrial genetic code. This approach preserves codon structure throughout the alignment and is equivalent to amino acid-guided alignment in its treatment of reading frame integrity. Frameshift characters introduced by MACSE were replaced with standard gap characters prior to downstream analyses. Ribosomal RNA genes (rrnS and rrnL) were aligned separately using MUSCLE with default settings, as this approach is appropriate for structural RNA sequences that lack codon structure. All alignments were trimmed using trimAl with the '-automated1' setting, which is optimized for maximum likelihood analyses. The trimmed alignments were then concatenated into the three multi-locus datasets described in the Methods.

Reviewer #3: "A mitochondrial genome represents a single maternally inherited linkage unit and cannot by itself represent the species tree. The authors should avoid overinterpreting the mitochondrial topology, especially for higher-level relationships within Accipitriformes. The phrase "basal taxa of Accipitriformes" should also be avoided... A more appropriate wording would be "early-diverging lineages within Accipitriformes, including Pandionidae and Sagittariidae.""

Response: We have replaced "basal" with "early-diverging" throughout the manuscript when referring to extant lineages (e.g., "early-diverging lineages within Accipitriformes, including Pandionidae and Sagittariidae," per the reviewer's suggested phrasing), and have added a caveat noting that the mitochondrial phylogeny reflects a single maternally inherited locus.

Reviewer #3: "The reported extra cytosine at position 174 of the nad3 gene is interesting. However, the authors should clearly explain how this insertion was handled during translation, sequence alignment, and dN/dS analysis. Since this insertion may cause an apparent frameshift but is known in birds and may involve translational frameshifting or RNA editing, it is important to clarify whether the insertion was retained, removed, or manually corrected in each downstream analysis."

Response: The extra cytosine at position 174 was manually corrected in each downstream analysis.

Reviewer #3: "The authors used SLAC/Datamonkey to calculate Ka/Ks or dN/dS values and found that all mitochondrial PCGs are under purifying selection... A single overall dN/dS value per gene across Accipitridae or Accipitriformes only indicates general conservation; it does not support claims regarding environmental adaptation... Otherwise, this section should be presented as a descriptive comparison of mitochondrial gene conservation."

Response: We have revised this section to be more descriptive and less speculative regarding their involvement in environmental adaptation.

Reviewer #3: "The manuscript repeatedly suggests that these mitogenomes can provide critical insights for genetic diversity monitoring and conservation management... The authors should state more cautiously that these mitogenomes provide reference sequences for future population genetic, phylogeographic, and conservation genomic studies."

Response: We have already revised the Conclusion and relevant Discussion passages which now state that the assembled mitogenomes "provide reference sequences that may support future population genetic, phylogeographic, and conservation genomic studies," and the closing sentence advocating direct conservation-strategy application has been removed.

Reviewer #3: "Fig. 3 is difficult to interpret in its current form... The authors should either redesign Fig. 3 with clearer ring labels and a more informative legend, or consider replacing it with a more interpretable visualization method."

Response: We have redesigned Figure 3 to focus on the CR/pseudo-CR specifically, with taxa grouped into four labeled categories (N. pinskeri, Other Nisaetus, Other Aquilinae, Other Accipitridae), spaced groupings, and an expanded caption.

Minor Comments

Reviewer #3: "The abstract should avoid overly strong phrases such as "critical insights" unless the conclusions are directly supported by the analyses."

Response: We have revised the abstract to remove "critical insights" and similar overstated phrasing, consistent with the Discussion/Conclusion revisions described above.

Reviewer #3: "Please ensure consistency in the reported length of the N. cirrhatus mitogenome. The abstract reports 17,873 bp, whereas the results section reports 17,874 bp."

Response: We have corrected this and report 17,874 bp for the N. cirrhatus mitogenome

Reviewer #3: "The phrase "family Accipitriformes" should be corrected, as Accipitriformes is an order, not a family."

Response: Corrected to "family Accipitridae”

Reviewer #3: ""itsadaptability" appears to lack a space."

Response: Corrected to "its adaptability."

Reviewer #3: "The attempted assembly of the Nisaetus floris dataset and its subsequent identification as likely Aquila fasciata are potentially important, but this part is currently unclear. The authors should clarify whether this re-identification is part of the present study and, if so, what evidence supports it... The authors should also state whether the N. floris dataset was excluded from all downstream analyses and whether this potential misidentification has been reported to the relevant database or museum collection."

Response: During mitogenome assembly of the dataset associated with the draft genome ASM2758013v1, the resulting sequence was consistently recovered as sister to Aquila fasciata rather than to any Nisaetus taxon. We then examined the associated BioSample metadata and voucher specimen (MCZ Orn 261829, Museum of Comparative Zoology, Harvard University), which has a documented history of being reclassified from Nisaetus floris to Aquila fasciata. Based on this evidence, we concluded that the specimen was likely mislabeled. We have also clarified that the ASM2758013v1 dataset was excluded from all downstream phylogenetic analyses presented in this study. Finally, we now explicitly state that the potential misidentification has not yet been formally reported to the NCBI.

Response to Reviewer #4

Reviewer #4: "The manuscript reports the length of N. cirrhatus as 17,873 bp in the abstract and conclusion, but 17,874 bp in the Results and in Figure 1. Please reconcile this discrepancy throughout the manuscript."

Response: We have corrected this and report 17,874 bp for the N. cirrhatus mitogenome

Reviewer #4: "In the Results, the N. pinskeri mitogenome is described as containing 13 PCGs, 20 tRNAs, 2 rRNAs, one control region, and one pseudo-control region, whereas the Conclusion states that both mitogenomes consist of the typical avian set of 13 PCGs, 2 rRNAs, and 22 tRNAs. Please clarify the final annotated gene content and ensure that the Results, figures, and Conclusion are fully consistent."

Response: We have corrected the Results to show 22 tRNAs.

Reviewer #4: "The maximum-likelihood analyses were run with 100 bootstrap replicates only, which is relatively modest for a study making several topological comparisons across datasets."

Response: Phylogenetic analysis methodology is now overhauled. Node support was assessed using 1000 ultrafast bootstrap replicates (UFBoot2) and 1000 SH-aLRT replicates.

Reviewer #4: "The phylogenetic Methods section states that 'the protein model' with the lowest AIC/BIC was chosen, but the trees were then inferred using nucleotide substitution models such as GTR+G+I and TN93+G+I. Please clarify whether the analyses were performed on nucleotide alignments, amino-acid alignments, or both, and revise the model-selection description accordingly."

Response: Phylogenetic analysis methodology was overhauled. The original trees were reconstructed using IQ-TREE2 with a fully partitioned analysis framework. For the protein-coding gene (PCG) datasets, each gene was partitioned by codon position into first and second codon positions (pos12) and third codon position (pos3) as separate subsets. Ribosomal RNA genes (rrnS and rrnL) were each treated as a single partition. Model selection was performed independently for each partition using ModelFinder implemented in IQ-TREE2, with the best-fit substitution model selected under the Bayesian Information Criterion (BIC).

Reviewer #4: "Table 1 appears to contain a classification problem because Circaetus pectoralis is listed under 'Cathartidae,' which is inconsistent with the broader structure of the table and with the manuscript's own discussion of Circaetinae."

Response: We have corrected this to say Circaetinae.

Reviewer #4: "Please replace 'singular mitochondrial genes' with 'single mitochondrial genes,' which is the more natural term in this context."

Response: Corrected as suggested throughout the manuscript.

Attachment Submitted filename: Response to Reviewers.docx

# Peer review: Decision Letter 1

**DOI:** 10.1371/journal.pone.0360538.r003

**Authors:** James Lee Crainey

28 Sep 2026

The Mitochondrial Genomes of the Endemic and Endangered South Philippine Hawk-eagle Nisaetus pinskeri and other Nisaetus Hawk-eagles (Aves, Accipitriformes)

PONE-D-26-01162R1

Dear Dr. Perdon,

We’re pleased to inform you that your manuscript has been judged scientifically suitable for publication and will be formally accepted for publication once it meets all outstanding technical requirements.

Within one week, you’ll receive an e-mail detailing the required amendments. When these have been addressed, you’ll receive a formal acceptance letter and your manuscript will be scheduled for publication.

An invoice will be generated when your article is formally accepted. Please note, if your institution has a publishing partnership with PLOS and your article meets the relevant criteria, all or part of your publication costs will be covered. Please make sure your user information is up-to-date by logging into Editorial Manager at Editorial Manager® and clicking the ‘Update My Information' link at the top of the page. For questions related to billing, please contact billing support.

If your institution or institutions have a press office, please notify them about your upcoming paper to help maximize its impact. If they’ll be preparing press materials, please inform our press team as soon as possible -- no later than 48 hours after receiving the formal acceptance. Your manuscript will remain under strict press embargo until 2 pm Eastern Time on the date of publication. For more information, please contact onepress@plos.org.

Kind regards,

James Lee Crainey, Ph.D.

Academic Editor

PLOS One

Additional Editor Comments (optional):

Your reviewers are satisfied that your revised manuscript is suitable for publication in PLOS ONE. Congratulations for all your hard work.

Reviewers' comments:

Reviewer's Responses to Questions

**Comments to the Author**

Reviewer #4: All comments have been addressed

**********

2. Is the manuscript technically sound, and do the data support the conclusions?

Reviewer #4: Yes

**********

3. Has the statistical analysis been performed appropriately and rigorously?

Reviewer #4: Yes

**********

4. Have the authors made all data underlying the findings in their manuscript fully available?

The PLOS Data policy

Reviewer #4: Yes

**********

5. Is the manuscript presented in an intelligible fashion and written in standard English?

Reviewer #4: Yes

**********

Reviewer #4: The changes made in response to the previous review comments have strengthened the manuscript considerably, and I have no further substantive concerns.

**********

what does this mean?). If published, this will include your full peer review and any attached files.

If you choose “no”, your identity will remain anonymous but your review may still be made public.

**Do you want your identity to be public for this peer review?**  For information about this choice, including consent withdrawal, please see our Privacy Policy

Reviewer #4: **Yes:**  Dr. Dwi Sendi Priyono

**********

# Editor report: Acceptance letter

**DOI:** 10.1371/journal.pone.0360538.r004

**Authors:** James Lee Crainey

PONE-D-26-01162R1

PLOS One

Dear Dr. Saloma,

I'm pleased to inform you that your manuscript has been deemed suitable for publication in PLOS One. Congratulations! Your manuscript is now being handed over to our production team.

At this stage, our production department will prepare your paper for publication. This includes ensuring the following:

* All references, tables, and figures are properly cited

* All relevant supporting information is included in the manuscript submission,

* There are no issues that prevent the paper from being properly typeset

You will receive further instructions from the production team, including instructions on how to review your proof when it is ready. Please keep in mind that we are working through a large volume of accepted articles, so please give us a few days to review your paper and let you know the next and final steps.

Lastly, if your institution or institutions have a press office, please let them know about your upcoming paper now to help maximize its impact. If they'll be preparing press materials, please inform our press team within the next 48 hours. Your manuscript will remain under strict press embargo until 2 pm Eastern Time on the date of publication. For more information, please contact onepress@plos.org.

You will receive an invoice from PLOS for your publication fee after your manuscript has reached the completed accept phase. If you receive an email requesting payment before acceptance or for any other service, this may be a phishing scheme. Learn how to identify phishing emails and protect your accounts at https://explore.plos.org/phishing.

If we can help with anything else, please email us at customercare@plos.org.

Thank you for submitting your work to PLOS One and supporting open access.

Kind regards,

PLOS One Editorial Office Staff

on behalf of

Dr. James Lee Crainey

Academic Editor

PLOS One
